# Effects of multivitamin, mineral and n-3 polyunsaturated fatty acid supplementation on aggression among long-stay psychiatric in-patients: randomised clinical trial

**DOI:** 10.1192/bjo.2022.8

**Published:** 2022-02-03

**Authors:** Nienke J. de Bles, Nathaly Rius-Ottenheim, Johanna M. Geleijnse, Ondine van de Rest, Jan P.A.M. Bogers, Anke Schat, Henk L.I. Nijman, David van den Berg, Lucas Joos, Annelies van Strater, Tine de Ridder, Joost J. Stolker, Wilbert B. van den Hout, Albert M. van Hemert, Erik J. Giltay

**Affiliations:** Department of Psychiatry, Leiden University Medical Center, The Netherlands; Department of Psychiatry, Leiden University Medical Center, The Netherlands; Division of Human Nutrition and Health, Wageningen University & Research, The Netherlands; Division of Human Nutrition and Health, Wageningen University & Research, The Netherlands; Intensive Care Clinics, Mental Health Organization Rivierduinen, The Netherlands; Department of Psychology, University of the Arts London, UK; Behavioural Science Institute, Radboud University, The Netherlands; and Forensic Psychiatric Institute, Fivoor, The Netherlands; Department of Psychosis Research, Parnassia Psychiatric Institute, The Netherlands; and Department of Clinical Psychology, Amsterdam Public Health Research Institute, Vrije Universiteit, The Netherlands; Sector psychosezorg, PZ Bethaniënhuis, Belgium; Fit op Weg poli Spijkenisse, GGZ Delfland, The Netherlands; PZ Campus Amedeus, Zorggroep Multiversum, Belgium; High Intensive Care, GGZ Eindhoven, The Netherlands; Department of Biomedical Data Sciences, Leiden University Medical Center, The Netherlands; Department of Psychiatry, Leiden University Medical Center, The Netherlands; Department of Psychiatry, Leiden University Medical Center, The Netherlands

**Keywords:** Aggression, supplements, nutrition, psychiatric in-patients, n-3 polyunsaturated fatty acids

## Abstract

**Background:**

Aggression and violent incidents are a major concern in psychiatric in-patient care. Nutritional supplementation has been found to reduce aggressive incidents and rule violations in forensic populations and children with behavioural problems.

**Aims:**

To assess whether multivitamin, mineral and n-3 polyunsaturated fatty acid supplementation would reduce the number of aggressive incidents among long-stay psychiatric in-patients.

**Method:**

The trial was a pragmatic, multicentre, randomised, double-blind placebo-controlled study. Data were collected from 25 July 2016 to 29 October 2019, at eight local sites for mental healthcare in The Netherlands and Belgium. Participants were randomised (1:1) to receive 6-month treatment with either three supplements containing multivitamins, minerals and n-3 polyunsaturated fatty acid, or placebo. The primary outcome was the number of aggressive incidents, determined by the Staff Observation Aggression Scale – Revised (SOAS-R). Secondary outcomes were patient quality of life, affective symptoms and adverse events.

**Results:**

In total, 176 participants were randomised (supplements, *n* = 87; placebo, *n* = 89). Participants were on average 49.3 years old (s.d. 14.5) and 64.2% were male. Most patients had a psychotic disorder (60.8%). The primary outcome of SOAS-R incidents was similar in supplement (1.03 incidents per month, 95% CI 0.74–1.37) and placebo groups (0.90 incidents per month, 95% CI 0.65–1.19), with a rate ratio of 1.08 (95% CI 0.67–1.74, *P* = 0.75). Differential effects were not found in sensitivity analyses on the SOAS-R or on secondary outcomes.

**Conclusions:**

Six months of nutritional supplementation did not reduce aggressive incidents among long-stay psychiatric in-patients.

Aggression and violent incidents are highly prevalent in psychiatric in-patient care, varying from nine to 90 incidents per patient per year, depending on the type of ward.^1^^–^^3^ Although pharmacotherapy and psychotherapy may help to mitigate feelings of irritability, anger or overt aggression,^4^ clinical guidelines emphasise the need for additional treatment options.

## Nutritional psychiatry

Research is increasingly suggesting diet to be a modifiable factor affecting mood and behaviour, giving rise to the field of nutritional psychiatry.^6^ Essential nutrients, including lipids, amino acids, vitamins and minerals, play an important role in biological processes such as inflammation, oxidative stress, neuroplasticity, neurogenesis and synthesis of neurotransmitters, with the gut–brain axis acting as a potential mediating pathway.^7^^,^^8^ For example, vitamin B6, vitamin B12 and folate are crucial in the formation of neurotransmitters such as epinephrine, norepinephrine, γ-amino butyric acid and serotonin.^7^ In particular, a deficiency in serotonin seems to play a key role in aggressive behavior,^9^^,^^10^ considering that the deprivation of the amino acid tryptophan, the dietary precursor of serotonin, can induce aggressiveness,^11^ and the selective serotonin reuptake inhibitor fluoxetine has shown anti-aggressive effects in randomised trials among psychiatric patients.^12^^,^^13^ However, as essential nutrients act in synergistic combinations, broad-spectrum micronutrients are recommended in trials among psychiatric populations, rather than focusing on a single nutrient.^14^

## Previous findings

Previous literature has explored whether multivitamin, mineral and n-3 polyunsaturated fatty acid (PUFA) supplementation may help to reduce aggressive behaviour. Hitherto, researchers in this field have studied young male prisoners^15^^–^^18^ and children with behavioural problems,^19^^–^^21^ some of whom were diagnosed with autism spectrum disorder,^22^ attention-deficit hyperactivity disorder,^23^ conduct disorder and oppositional defiant disorder.^24^ In total, five out of six randomised controlled trials showed reductions in aggression, and there were 26–47% fewer aggressive-related incidents in the group receiving nutritional supplements compared with those receiving a placebo.^15^^–^^19^ In addition, one of these studies demonstrated that participants with the lowest nutrient concentrations seemed to have benefited the most from nutritional supplementation.^17^ Reductions in aggressive behaviour based on the number of disciplinary incidents were not found in a study among school-aged children.^21^ In the same study, however, an observer-rated scale was also included that did show a significant reduction in these behaviours in the intervention compared with the control group. Trials that assessed subjective feelings of aggression as an outcome showed consistent findings.^20^^,^^22^^–^^24^ Thus, nutritional supplementation may help to reduce aggression, but this needs to be confirmed in a sample of long-stay psychiatric in-patients.

Long-stay psychiatric in-patients are more at risk for nutrient deficiencies compared with the general population, because of the consumption of more energy-dense and nutrient-poor diets, insufficient outdoor activities and the potential detrimental effect of psychotropics (e.g. antipsychotics) on appetite, gastrointestinal function, the microbiome and (energy and micronutrient) metabolism.^25^^,^^26^ The overall poor quality dietary intake was also confirmed in a pilot study in a group of 21 patients from the target population (Supplementary Appendices 1 and 2 available at https://doi.org/10.1192/bjo.2022.8). Hence, it can therefore be hypothesised that psychiatric in-patients may likely benefit from nutritional supplementation.

## Aim and hypothesis

A randomised, double-blind placebo-controlled trial was initiated to determine the effectiveness of nutritional supplements in reducing aggressive incidents among long-stay psychiatric in-patients. We hypothesised that nutritional supplementation would reduce aggressive incidents, feelings of aggression and affective symptoms, and would increase patient quality of life.

## Method

### Design

This pragmatic, multicentre, randomised, double-blind, placebo-controlled intervention trial was coordinated in the Department of Psychiatry at the Leiden University Medical Centre (LUMC). Participants were recruited between 25 July 2016 and 29 October 2019, from eight local sites for mental healthcare in The Netherlands and Belgium. Data collection took place at the ward where the participants resided. Participants received a small financial compensation (€2.50) for completing each assessment. Written informed consent was obtained from all participants, in some cases from a relative or legal representative, where appropriate. All procedures contributing to this work complied with the ethical standards of the relevant national and institutional committees on human experimentation and with the Helsinki Declaration of 1975, as revised in 2008. The trial protocol (Supplementary Appendix 3) was approved by the Medical Ethical Committee of the LUMC, under reference number P14.332, and the study followed the Consolidated Standards of Reporting Trials (CONSORT) reporting guideline. The trial was registered at ClinicalTrials.gov (identifier NCT02498106).

### Participants

Inclusion criteria were age ≥18 years and expected to reside at a facility for long-term psychiatric in-patient care for at least 6 months, irrespective of their specific psychiatric disorder. Exclusion criteria were pregnancy, breastfeeding, contraindication for nutritional supplements, expected discharge or transfer within 8 weeks, restrictions against the consumption of pork gelatine and continuous use of other nutritional supplements within the preceding 8 weeks (exceptions included vitamin B1 and vitamin D, which are mostly prescribed to prevent complications of alcoholism or to treat low vitamin D plasma levels in Northern countries, respectively, and which entailed no health risks in combination with this study's supplements).

### Intervention

A 2-week placebo run-in phase was followed by a 6-month nutritional intervention, during which participants received a daily dose of two capsules containing multivitamins and minerals and one capsule containing n-3 PUFA (i.e. eicosapentaenoic acid and docosahexaenoic acid;^27^ Supplementary Appendix 4). The control group received three placebo capsules per day, containing the neutral n-9 oleic acid. The n-3 PUFA content of both nutritional supplements and placebo capsules was checked during follow-up and showed no noticeable decay. Intervention costs were estimated at €393 per patient year, of which 80% was for the supplements and 20% was for distribution (assuming 30 s of nursing staff time per patient per day, at €26 per hour; Supplementary Appendix 5).

### Randomisation

Participants were randomised in a 1:1 ratio, using blocks of 12 participants, and were stratified by gender and ward type (open or closed). At the end of the study, participants and nurses were asked whether they thought the participant had received supplements or placebo, to check whether blinding had been successful.

### Measurements

#### Primary outcome variable

The primary outcome in this study was the number of aggressive incidents registered, as determined by the Staff Observation Aggression Scale – Revised (SOAS-R), created originally for use in in-patient psychiatric wards.^28^ The SOAS-R is a quick and easy-to-use tool, which comprises five columns recording provocation, means used, the target, consequences and measures taken to stop aggression. Each time an aggressive incident occurred, nursing staff were expected to complete the SOAS-R. Aggression was defined as ‘any verbal, nonverbal, or physical behaviour that was threatening (to self, others, or property) or [any] physical behaviour that actually did harm (to self, others, or property)’.^28^^,^^29^ The SOAS-R total severity score ranges from 0 to 22 points, with scores of 0–7 indicating mild, 8–15 indicating moderate and 16–22 indicating severe severity.^30^ Severity of an incident was also judged on a 100-mm visual analogue scale, ranging from 0 (not severe at all) to 100 (extremely severe). Results on the psychometric properties of the SOAS-R indicate fair to good interrater reliability, with an intraclass correlation of 0.96,^31^ reported Cohen's kappas of between 0.61 and 0.74^32^^–^^34^ and a Pearson's product-moment *r* between independent raters of 0.87.^32^ The SOAS-R severity scores indicate significant concurrent validity.^1^

#### Secondary outcome variables

The 11-item Social Dysfunction and Aggression Scale (SDAS)^35^ was completed by nurses at baseline and after 2 weeks, 2 months and 6 months. In our sample, the intraclass correlation was 0.76, suggesting moderate stability over time. It is recommended to use the SDAS in conjunction with the SOAS-R, as the former is more sensitive to measure more subtle aggression incidents.^36^ Additionally, patients were asked to complete several questionnaires at baseline, 2 months and 6 months: A Dutch version of the shortened 12-item Aggression Questionnaire,^37^^,^^38^ the 26-item World Health Organization Quality of Life^39^^,^^40^ and the abbreviated 25-item version of the Comprehensive Psychopathological Rating Scale (CPRS).^41^^,^^42^ The CPRS included the ten-item Montgomery–Åsberg Depression Rating Scale,^43^ the ten-item Brief Anxiety Scale^44^ and a five-item inhibition subscale.

#### Other variables

Non-fasting blood samples were collected to determine nutritional status and to monitor adherence in those who consented to blood collection (in 82.6% of participants in The Netherlands). Belgian institutions did not collect blood because of pragmatic reasons, as samples were transported through the regular postal service. Blood samples were collected only if a participant agreed to a venipuncture. Two tubes were collected (one serum separator and one ethylenediaminetetraacetic acid (EDTA) tube) before and after the trial, by competent nurses appointed in each institution. Subsequently, blood samples were sent to the LUMC within 24 h, through the regular postal service. The samples were stored at −80°C until analysis. Samples were analysed for vitamins A (retinoids), E (tocopherol), B12 (cobalamin) and D (calciferol), folic acid and iron in blood serum. Vitamin B1 (thiamine), vitamin B6 (pyridoxine) and a fatty acid spectrum to yield n-3 fatty acid levels (alpha-linolenic acid, eicosapentaenoic acid and docosahexaenoic acid ) were analysed in EDTA blood samples. Blood level assessments are described in detail in the Supplementary Material.

Sociodemographic covariates were age, gender, level of education (categorised into low (primary education, lower secondary education), medium (upper secondary education, post-secondary non-tertiary education) and high (tertiary education, Bachelor's, Master's, doctoral)), marital status (never married, ever married (married, widow/widower or divorced)), ancestry (European, non-European ancestry), smoking (yes, no), any use of recreational drugs (never, ever, current (past month)) and use of alcohol (>14 units per week). Body mass index was calculated based on measured height and weight. Ward type (open, closed), primary diagnosis and medication use were obtained from the treating psychiatrist.

### Statistical analyses

The sample size was based on results of previous literature.^15^^–^^17^^,^^19^^,^^45^^,^^46^ We assumed a conservative delta of 26% reduction in the number of incidents, whereby incidents are modelled according to a Poisson distributed random variable.^47^ Assuming 80% power, *α* = 0.05 and an overall rate of 4, a final total sample size of 132 was required to be able to reject the null hypothesis. Assuming a drop-out rate of 25%, we aimed to include at least 166 patients.

Sociodemographic and clinical characteristics were summarized per allocation, using independent samples *t*-test and *χ*^2^-test. Micronutrient status was assessed with linear mixed models. The frequency of aggressive incidents was presented as the back-transformed geometric mean number of incidents per month. Negative binomial regression analyses were performed to analyse the number of aggressive incidents, as overdispersion was anticipated. An offset was used to take the log number of days that a patient participated in the trial into account. We applied triple masking, which ensured that the treatment was unknown to the participants and to the nurses and physicians, as well as the epidemiologist (J.M.G.) who analysed the effect on the primary outcome but was not part of the coordinating centre.

To investigate the trend of the incidence rate ratio, a negative binomial regression was performed for each month separately, plotted over time. Incidents were studied in total and individually, according to their level of severity and type. Sensitivity analyses were performed in subgroups excluding patients with an extreme number of incidents (i.e. either zero incidents per month or more than ten incidents per month), adjusting for baseline SDAS. *Post hoc* subgroup analyses were performed for sociodemographic and clinical variables. Differences between the randomised groups on the secondary outcomes were performed following intention-to-treat (ITT) analysis, using multilevel regression (mixed) models. In the case of missing data, we used last observation carried forward for the ITT analyses. *χ*^2^-Tests were performed to check whether participants and nurses gave the correct answer more often than expected by chance, excluding the ones who gave the answer ‘I do not know’. *χ*^2^-Tests were also performed to compare the number of side-effects among the randomised groups. A two-tailed significance level of *P* < 0.05 was considered statistically significant. Negative binomial regression analyses were performed with R software within RStudio (R version 3.6.0 for macOS; R Foundation for Statistical Computing, Vienna, Austria, 2016; https://www.R-project.org/) and the main package MASS (version 7.3). All other analyses were performed with IBM SPSS statistical software (version 25, IBM Corp, 2017, IBM SPSS Statistics for Windows).

## Results

**Fig. 1:**
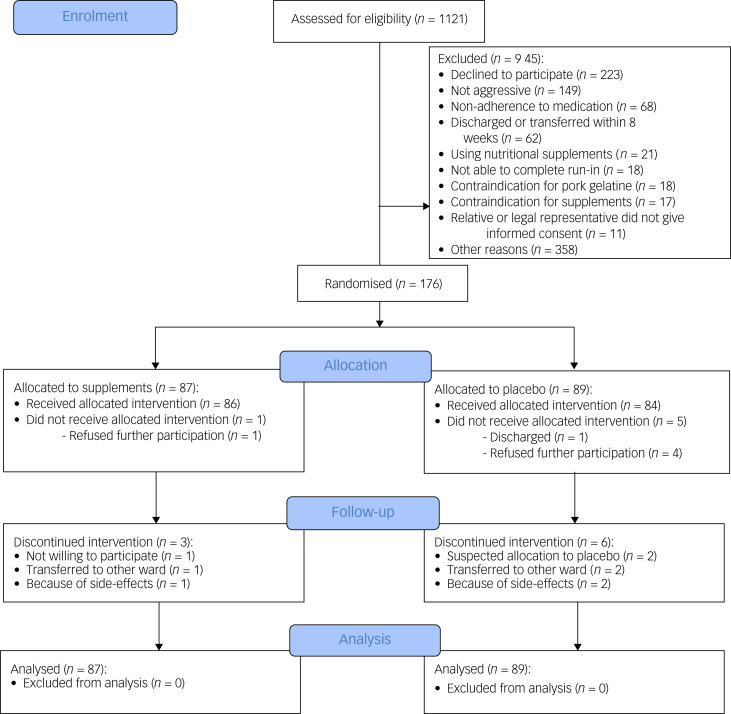
Consolidated Standards of Reporting Trials flow diagram of participants through the study.

We assessed 1121 patients for eligibility and excluded 945 (Fig. 1). The high number of excluded patients could be explained by the fact that treating psychiatrists regularly did not give permission to contact certain patients because it might disturb their treatment plan or therapeutic relationship (*n* = 358). In total, 176 participants were randomised into the trial (supplements, *n* = 87; placebo, *n* = 89), most of whom had a psychotic disorder (60.8%). The mean age of the participants was 49.3 years (s.d. 14.5), and 64.2% were male. No significant demographic or clinical group differences were observed at baseline (Table 1).

**Table 1:** Baseline characteristics of study participants (*N* = 176)

	Supplements (*n* = 87)	Placebo (*n* = 89)	*P*-value
Demographics			
Male gender, *n* (%)	54 (62.1%)	59 (66.3%)	0.56
Age in years, mean (s.d.)	49.1 (14.2)	49.4 (14.8)	0.88
BMI, kg/m^2^, mean (s.d.)	28.9 (6.0)	28.5 (7.9)	0.73
Closed ward, *n* (%)	56 (64.4%)	55 (61.8%)	0.72
Never married, *n* (%)	63/82 (76.8%)	69/85 (81.2%)	0.49
Education low, *n* (%)	49/79 (62%)	47/79 (59.5%)	0.74
European ancestry, *n* (%)	71 (81.6%)	70 (78.7%)	0.62
Current smoker^a^, *n* (%)	61/86 (70.9%)	64/86 (74.4%)	0.61
Alcohol ≥14 units per week^a^, *n* (%)	9/76 (11.8%)	7/82 (8.5%)	0.49
Recreational drugs^b^, *n* (%)	46/85 (54.1%)	48/85 (56.5%)	0.76
Clinical data			
Primary diagnosis, *n* (%)			0.97
Psychotic disorder	52 (59.8%)	55 (61.8%)	
Mood disorder	8 (9.2%)	9 (10.1%)	
Personality disorder	9 (10.3%)	9 (10.1%)	
Other	18 (20.7%)	16 (18.0%)	
Medication use			
Antipsychotics	76/80 (95.0%)	70/80 (87.5%)	0.09
FGA	42/80 (52.5%)	33/80 (41.3%)	0.15
SGA	60/80 (75.0%)	54/80 (67.5%)	0.29
Antidepressants	31/80 (38.8%)	31/80 (38.8%)	1.00
Benzodiazepines	61/80 (76.3%)	67/80 (83.8%)	0.24
Mood stabilisers	38/80 (47.5%)	35/80 (43.8%)	0.63

Data are number of participants (with percentages in parentheses) or means (with s.d. in parentheses). BMI, body mass index; FGA, first-generation antipsychotics; SGA, second-generation antipsychotics.

a. Based on the past month.

b. Ever used.

### Protocol adherence

In total, 114 participants agreed to blood sampling (82.6% of 138 participants from The Netherlands) at baseline, end-point or both. Expected increases were found in the intervention compared with placebo groups, which were statistically significant for vitamin B6 (*P* = 0.005), folic acid (*P* < 0.001), vitamin B12 (*P* = 0.04), vitamin E (*P* = 0.02), eicosapentaenoic acid (*P* < 0.001) and docosahexaenoic acid (*P* < 0.001; Supplementary Appendix 6).

### Primary outcome measures

**Fig. 2:**
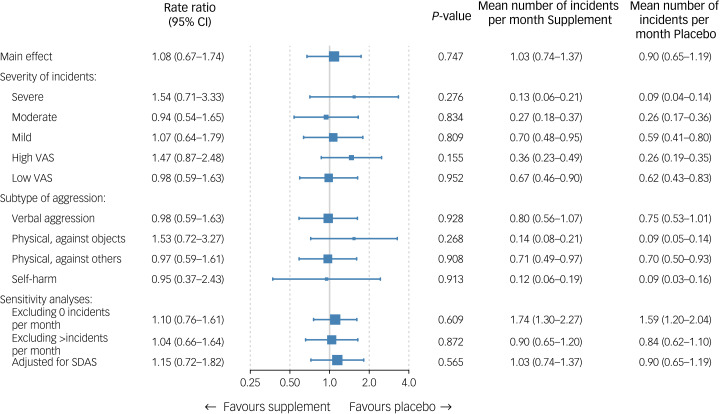
Effectiveness analyses on primary outcome. The mean is the geometrical mean number of incidents per month. Severe: 16–22, moderate: 8–15, mild: 0–7; high VAS: ≥5, low VAS: <5. SDAS, Social Dysfunction and Aggression Scale; VAS, visual analogue scale.

Figure 2 presents the main outcomes. The primary outcome of SOAS-R incidents was similar in those assigned to supplements (1.03 incidents per month, 95% CI 0.74–1.37) and placebo (0.90 incidents per month, 95% CI 0.65–1.19), with a rate ratio of 1.08 (95% CI 0.67–1.74, *P* = 0.75). Also, no significant effects were found according to the severity or type of aggressive incidents. Sensitivity analyses in subgroups according to the number of incidents (i.e. either zero or more than ten incidents per month) did not influence these results, which also applies to the analysis adjusting for baseline SDAS score. Supplementary Appendix 7 shows the incidence rate ratio per month during the 6-month intervention period. The geometrical mean number of incidents in a curve showed that during the first 2 months after the start of the intervention, patients in the intervention group had a slightly higher (but not significant) rate of aggressive incidents compared with those receiving placebo. As shown in Supplementary Appendix 8, subgroup analyses showed no evidence for effect modification by other variables, except for antipsychotic use. In the small subgroup of participants not using antipsychotics (*n* = 14), supplements showed a beneficial effect compared with placebo (*P* = 0.004).

### Secondary outcome measures

As seen in Table 2, an ITT approach showed that nutritional supplementation did not significantly affect any of the secondary outcomes. In detail, no differential effects for supplements versus placebo were found for either self- and observer-rated aggression, quality of life, depression severity, anxiety severity or inhibition.

**Table 2:** Intention-to-treat analyses of the effectiveness on secondary outcomes

		*n*	Baseline	2 weeks	2 months	6 months	Interaction: score × time	
							Test	*P*-value
SDAS	Supplements	84	9.2 (0.9)	8.2 (0.9)	7.9 (0.8)	7.7 (0.8)	F(d.f. 1) = 0.016	0.90
	Placebo	84	9.6 (0.9)	8.0 (0.7)	8.4 (0.8)	7.8 (0.7)		
Aggression Questionnaire	Supplements	84	30.4 (1.2)	–	28.7 (1.2)	30.1 (1.2)	F(d.f. 1) = 0.576	0.45
	Placebo	84	32.0 (1.1)	–	30.9 (1.0)	30.8 (1.0)		
Anger	Supplements	82	7.5 (0.4)	–	7.0 (0.4)	7.2 (0.4)	F(d.f. 1) = 0.006	0.94
	Placebo	84	8.2 (0.4)	–	7.8 (0.4)	7.9 (0.3)		
Hostility	Supplements	84	9.0 (0.4)	–	8.6 (0.4)	8.8 (0.4)	F(d.f. 1) = 0.319	0.57
	Placebo	82	9.5 (0.4)	–	9.6 (0.4)	9.0 (0.4)		
Physical aggression	Supplements	82	7.3 (0.4)	–	6.8 (0.4)	7.0 (0.4)	F(d.f. 1) = 0.052	0.82
	Placebo	83	7.1 (0.4)	–	6.8 (0.3)	6.8 (0.4)		
Verbal aggression	Supplements	81	6.4 (0.4)	–	6.4 (0.4)	6.8 (0.4)	F(d.f. 1) = 0.958	0.33
	Placebo	82	7.4 (0.3)	–	7.0 (0.3)	7.4 (0.3)		
WHO-QOL	Supplements	77	87.4 (2.0)	–	85.2 (2.1)	85.8 (2.0)	F(d.f. 1) = 0.596	0.44
	Placebo	79	83.5 (1.9)	–	83.6 (1.8)	84.0 (1.8)		
Physical health	Supplements	78	13.2 (0.4)	–	13.0 (0.4)	13.2 (0.4)	F(d.f. 1) = 0.001	0.98
	Placebo	78	12.6 (0.4)	–	12.7 (0.3)	12.8 (0.3)		
Psychological health	Supplements	78	12.6 (0.4)	–	12.7 (0.4)	12.4 (0.4)	F(d.f. 1) = 0.952	0.33
	Placebo	77	12.4 (0.4)	–	12.4 (0.3)	12.7 (0.4)		
Social relationships	Supplements	78	12.9 (0.4)	–	12.6 (0.4)	12.6 (0.5)	F(d.f. 1) = 0.687	0.41
	Placebo	74	12.6 (0.4)	–	12.5 (0.4)	12.9 (0.4)		
Environment	Supplements	78	14.4 (0.3)	–	13.8 (0.4)	14.1 (0.3)	F(d.f. 1) = 0.105	0.75
	Placebo	79	13.7 (0.3)	–	13.8 (0.3)	13.5 (0.3)		
MADRS	Supplements	69	13.0 (1.3)	–	12.2 (1.3)	12.5 (1.2)	F(d.f. 1) = 0.021	0.89
	Placebo	71	13.3 (1.3)	–	13.0 (1.3)	13.2 (1.2)		
BAS	Supplements	69	12.0 (1.0)	–	10.7 (0.9)	11.5 (0.9)	F(d.f. 1) = 0.033	0.86
	Placebo	68	11.8 (0.9)	–	11.6 (0.8)	11.6 (0.8)		
Inhibition Scale	Supplements	70	4.7 (0.5)	–	4.0 (0.5)	4.1 (0.4)	F(d.f. 1) = 0.462	0.50
	Placebo	71	4.7 (0.5)	–	4.6 (0.5)	4.6 (0.5)		

Data are means (with s.e. in parentheses). SDAS, Social Dysfunction and Aggression Scale, observer rated; WHO-QOL, World Health Organization Quality of Life; MADRS, Montgomery–Åsberg Depression Rating Scale; BAS, Brief Anxiety Scale.

### Blinding

Blinding was successful among participants, who guessed correctly whether they had been taking the supplements or placebo no more frequently than incorrectly (*P* = 0.44). Nurses who distributed the supplements, however, more frequently guessed the randomised condition of the participants correctly (*n* = 38 out of 55; 69.1% correct; *P* = 0.005). Still, the majority of both participants (*n* = 48 out of 116; 41.4%) and nurses (*n* = 75 out of 130; 57.7%) answered with ‘I do not know’.

### Adverse effects

At least one side-effect was reported by 15 out of 73 patients in the control group (20.5%) and 17 out of 74 patients in the intervention group (23.0%; *P* = 0.72; see Supplementary Appendix 9). Burping (*P* = 0.049) and rash (*P* = 0.04) were significantly more frequently reported by participants in the intervention group compared with the control group, with burping being the most frequently reported (11.1 *v.* 2.8%). In total, two participants died during the trial (supplements, *n* = 1; placebo, *n* = 1). The age of these participants was 55 and 83 years. Reasons of death were respiratory disease and cardiac arrest, respectively. This was confirmed by their physicians, who all judged that there were no causal relationships with the supplements.

## Discussion

Our findings provided no support for the effectiveness of multivitamin, mineral and n-3 PUFA supplementation in reducing the number of aggressive incidents among psychiatric in-patients during a 6-month intervention. *Post hoc* analyses according to the severity or type of aggressive incidents corroborated this conclusion. No differences were found between the randomized groups regarding the secondary outcomes, including self- and observer-rated aggression, quality of life and affective symptom severity.

The current study is the first to investigate the effect of nutritional supplements in in-patients suffering from chronic psychiatric disorders. These patients are often not included in clinical trials, leading to a lack of evidence for effective care and treatment.^48^ In previous trials that investigated the effect of nutritional supplements on aggressive incidents, patients with psychosis were often excluded^20^^,^^21^^,^^23^^,^^24^ or no information on the use of psychotropic medication was given.^15,19^ Our sample included participants with psychotic disorders (60.8%), a vast majority of whom were using antipsychotics (91.2%). The extensive use of antipsychotics in our population may have led to a ceiling effect, as antipsychotics are prescribed to mitigate agitation and aggression,^49^ creating a situation in which no additional effect of a nutritional intervention could be found. Additionally, adverse effects of antipsychotics comprise gastrointestinal and metabolic side-effects, which may result in dysbiosis,^26,50^ the disruption of the bacterial species of the gut microbiota, which could potentially adversely affect mood and behaviour through the gut–brain axis.^8^ Note that an exploratory analysis of the subsample of patients with psychosis who did not use antipsychotic agents in the current study did suggest a reduction of incidents among the patients with psychosis who had taken the nutritional supplements. Furthermore, an exploratory trial including acute patients with schizophrenia treated with antipsychotic medication found no effect of n-3 PUFA supplementation on hostility compared with the control group.^51^ In addition, the incidents of the patients with psychosis in the current study may comprise different forms of aggression than those expressed by participants in previous trials, such as aggression resulting from the nature of their psychiatric disorders, like paranoid delusions.^52^ Moreover, aggressive behaviours in psychiatric patients may be masked by the complex interaction of different causal factors.

Besides the different study populations, there may be several other explanations for the discrepant findings of the current study with previous trials showing beneficial effects.^15–19^ The current study was the first to investigate the intervention among older patients, with a mean age of 49.3 years, whereas the mean age of participants from earlier forensic and youth trials ranged from 5 to 25 years. This is important as supplementation may have different effects across the lifespan, depending on the stages of brain development.^53^ Second, although some studies used a similar combination of multivitamins, minerals and n-3 PUFA,^15,16,20,21,24^ many others did not include n-3 PUFA in their active treatment arm;^17–19,22,23^ n-3 PUFA is known to be involved in brain structure and function.^7^ Dosages in prior studies varied in their recommended daily allowance (RDA), but two studies used substantially higher dosages.^23^ One of these two studies was a recently published three-arm trial that demonstrated that participants in the RDA group showed significantly less serious rule violations, whereas the participants in the higher-dose supplement group did not, compared with the placebo group.^18^ In our trial, we used relatively high dosages of B vitamins because a meta-analysis showed increased beneficial effects on perceived stress and hostility in the trials that used higher doses of B vitamins.^54^ Furthermore, the duration of the exposure in our trial was relatively long (6 months) compared with previous trials, which had a median intervention period of 3 months (ranging from 70 to 142 days). The longer exposure used in our study ensured the minimal duration of supplementation to establish equilibration of n-3 PUFA into different organs, including the brain.^55^ Our sample size of 176 participants was comparable to earlier trials, with a median number of randomised participants of 209 (ranging from 62 to 468).

In line with our primary outcome, we found no evidence for improvements on self- and observer-rated aggression questionnaires. However, previous trials that found significant aggression reductions also did not observe improvements on self- and observer-rated aggression questionnaires. This suggests that supplements affect objectively observed aggressive incidents more than subjectively perceived aggressive feelings and behaviour.^15,18,20,24^

Some limitations need to be discussed. First, we were obliged to be explicit toward potential participants about our aim to reduce aggressive incidents. As a consequence, patients, especially the more aggressive ones, were often not willing to participate.^56^ Second, nurses had to complete the SOAS-R in addition to writing up a description of the event in the patient records. Because of the high workload in psychiatric care, some (milder) incidents could have been missed. However, this phenomenon likely occurred in equal proportions in both randomised groups, and no beneficial effect was found for severe incidents, which were unlikely to be missed. In addition, using two different instruments (i.e. the incident-based SOAS-R and the SDAS based on the preceding week) simultaneously in the recording of aggressive behaviour gave us more complete information on the effects of the intervention.^36^ Third, although blinding was effective in participants, retrospectively, nurses often guessed the participant condition correctly. Fourth, as included participants were diagnosed with diverse psychiatric disorders, we cannot exclude the possibility that nutritional supplements would be beneficial within one specific kind of psychiatric disorder. Yet, subgroup analyses performed according to the diagnosis (i.e. psychotic disorder versus other) showed no evidence for effect modification. Fifth, participants from Belgium could not provide blood samples because of logistical reasons. Finally, no information was gathered about dietary habits, as most of our participants suffered from mental disorders characterised by disruptions in thought processes and were therefore less likely to complete food frequency questionnaires accurately.

In summary, this is the first randomised controlled trial that studied the effect of nutritional supplementation among long-stay psychiatric in-patients. Despite some promising effects of nutritional supplementation on aggressive incidents found in previous studies, we found no evidence of effect in chronically ill psychiatric in-patients.

## Data Availability

The data that support the findings of this study are available from the corresponding author, N.J.B., upon reasonable request.

## Supplementary material

For supplementary material accompanying this paper visit http://doi.org/10.1192/bjo.2022.8. click here to view supplementary material
