# Supplementary material for: Effects of multivitamin, mineral and n-3 polyunsaturated fatty acid supplementation on aggression among long-stay psychiatric in-patients: randomised clinical trial
Source: BJPsych Open. 2022 Feb 3;8(2):e42. doi: 10.1192/bjo.2022.8 (PMC8867900; doi:10.1192/bjo.2022.8)
Supplement: Supplementary file 1 [file bjosup.zip › S2056472422000084sup001.docx]

# Members of the Diet and Aggression Study

- Executive Committee: E.J. Giltay (principal investigator), N. Rius-Ottenheim (coordinating investigator), N.J. de Bles (trial coordinator), A.M. van Hemert (sponsor) – all at the Department of Psychiatry, Leiden University Medical Center, Leiden; J.M. Geleijnse (nutritionist, epidemiologist, Division of Human Nutrition and Health, Wageningen University & Research, Wageningen), O. van de Rest (nutritionist, Division of Human Nutrition and Health, Wageningen University & Research, Wageningen).
- Grant application: A. Schat, A.A.M. Hubers, and E.J. Giltay (Department of Psychiatry, Leiden University Medical Center, Leiden).
- Randomization: M.A. van der Hoorn (Department of Psychiatry, Leiden University Medical Center, Leiden), G.A.M. Corton (Department of Psychiatry, Leiden University Medical Center, Leiden).
- Participating mental health care institutions: Mental Health Organization Rivierduinen, Oegstgeest (J.P.A.M. Bogers, J.T.M. Remmerswaal); Fivoor, Den Dolder (H.L.I. Nijman, C.G.B. Taguba); Parnassia Psychiatric Institute, The Hague (D. van den Berg, N.M. van Gent); PZ Bethaniënhuis, Zoersel, Belgium (L. Joos, W. Peinen); GGZ Delfland, Delft (A. van Strater, R. Rozenstraten); GGZ Eindhoven, Eindhoven (J.J. Stolker, K. Weekers); Zorggroep Multiversum, Mortsel, Belgium (T. de Ridder, L. Van Belle); GGZ Centraal, Ermelo (N. Meijwaard).
- Data collection: A.W.P. Hazewinkel, K. Recourt, S.N.W. Setroikromo, M. Brobbel, S.E. Molengraaf, E.V. van Wijk, K.A.D. van Hövell, J. Bouwmeester, N.G. Vlug, I.M. Blok, A. den Hertog, X.D. Veldhuis, J.H.J. Plettenburg, L.E.H. Christiaansen, J.E.H. van der Does, N.D. Hubert, Y.M.A. Bos, M.F. Frölich, S. van der Zwaan.
- Independent physician: M.S. van Noorden (Department of Psychiatry, Leiden University Medical Center, Leiden).
- Cost-effectiveness analysis: W.B. van den Hout (Department of Biomedical Data Sciences, Leiden University Medical Center, Leiden)
- Laboratories: Division of Human Nutrition and Health, Wageningen University & Research, Wageningen (M.G.J. Balvers, P. Versloot); Department of Clinical Chemistry and Laboratory Medicine, Leiden University Medical Center, Leiden (R.C. van Wissen, J.A. Bakker, R.N. Malhoe Mishre-Lalai).
- Trial supplements: MCO Health, Almere (D. de Bruine, E. Alberts).
