# Supplementary material for: Effects of multivitamin, mineral and n-3 polyunsaturated fatty acid supplementation on aggression among long-stay psychiatric in-patients: randomised clinical trial
Source: BJPsych Open. 2022 Feb 3;8(2):e42. doi: 10.1192/bjo.2022.8 (PMC8867900; doi:10.1192/bjo.2022.8)
Supplement: Supplementary file 1 [file bjosup.zip › S2056472422000084sup002.pdf]

Supplementary Material

Supplementary Material 1 Characteristics of psychiatric inpatients versus controls.

Supplementary Material 2 Comparison of dietary intake of food groups and micro(nutrients) in psychiatric inpatients versus controls.

Supplementary Material 4 Doses of previous studies including the current study.

Supplementary Material 5 Average costs (in €) per patient year, by randomization group.

Supplementary Material 6 Changes in micronutrient levels between supplementation and placebo.

Supplementary Material 7 Effect on aggressive incidents over time during 6 months of supplementation.

Supplementary Material 8 Subgroup analyses of the effect of supplementation on aggressive incidents according to demographic and clinical data.

Supplementary Material 9 Adverse effects in both groups.

Supplementary Material 1 Characteristics of psychiatric inpatients versus controls.

	Psychiatric inpatients (n=21)	Controls (n=38)	*P* value[a]
Age in years, mean (*SD*)	40.0 (13.1)	46.7 (16.3)	0.11
Male gender, no (%)	14 (66.7%)	18 (47.4%)	0.12
Higher education, no (%)	5 (23.8%)	30 (78.9%)	< 0.001
BMI (kg/m^2), mean (*SD*)	26.9 (7.8)	24.4 (3.6)	0.11
Dutch Nationality, no (%)	14 (66.7%)	38 (100%)	< 0.001
Alcohol ≥ 14 U/wk, no (%)	1 (4.8%)	12 (31.6%)	0.02
Current smoker, no (%)	21 (100%)	15 (39.5%)	< 0.001
Recreational drugs, no (%)	8 (38.1%)	0 (0%)	< 0.001
Unmarried, no (%)	21 (100.0%)	10 (26.3%)	< 0.001
Physically active, no (%)	5 (23.8%)	18 (47.4%)	0.07
Psychotropic use, no (%)	21 (100.0%)	0 (0.0%)	< 0.001

Note. Data are number of participants (with percentages in parentheses) or means (with standard errors in parentheses). BMI=Body Mass Index.

[a] Chi-square values have been computed for categorical variables, t-test for independent samples for interval variables.

Supplementary Material 2 Comparison of dietary intake of food groups and micro(nutrients) in psychiatric inpatients versus controls.

	Psychiatric inpatients (n=21)	Controls (n=38)	Adjusted Mean difference	*P* value[a]
Total caloric intake[b]	1700 (120.8)	2016 (90.7)	–316 (–620; –13)	< 0.001
Total protein, g	69.0 (2.9)	72.6 (2.1)	–3.71 (–11.4; 3.96)	0.34
Vegetable protein, g	31.9 (1.7)	30.9 (1.2)	1.00 (–3.34; 5.34)	0.65
Animal protein, g	37.1 (3.0)	41.7 (2.2)	–4.65 (–12.6; 3.27)	0.24
Total fat, g	62.3 (2.6)	63.7 (1.9)	–1.32 (–8.09; 5.45)	0.70
Saturated fatty acids, g	23.6 (1.2)	23.8 (0.8)	–0.19 (–3.23; 2.84)	0.90
Mono unsaturated fatty acids, g	20.7 (1.1)	22.4 (0.8)	–1.71 (–4.57; 1.15)	0.24
Poly unsaturated fatty acids, g	12.1 (0.9)	11.2 (0.7)	0.90 (–1.55; 3.36)	0.47
Trans fatty acids, g	1.22 (0.1)	1.48 (0.1)	–0.26 (–0.49; –0.03)	0.03
-Linolenic acid (ALA, mg)	0.98 (0.1)	0.94 (0.01)	0.04 (–0.13; 0.20)	0.68
Eicosapentaenoic acid (EPA, mg)	0.02 (0.02)	0.06 (0.01)	–0.04 (–0.08; 0.001)	0.056
Docosahexaenoic acid (DHA, mg)	0.04 (0.02)	0.10 (0.02)	–0.06 (–0.12; -0.004)	0.035
Cholesterol, mg	140 (12)	200 (8.6)	–60.8 (–92.2; –29.5)	< 0.001
Total carbohydrates, g	262 (7.9)	224 (5.6)	38.1 (17.6; 58.7)	< 0.001
Total mono and disaccharides, g	149 (6.8)	105 (4.8)	44.1 (26.4; 61.7)	< 0.001
Total polysaccharides, g	112 (6.0)	119 (4.3)	–6.32 (–21.9; 9.31)	0.42
Total dietary fibre, g	20.7 (1.3)	23.2 (0.9)	–2.53 (–6.00; 0.92)	0.15
Calcium (mg)	919 (78.5)	857 (56.1)	62.2 (–143; 267)	0.55
Iron (mg)	11.2 (0.5)	11.7 (0.3)	–0.50 (–1.71; 0.72)	0.41
Zinc (mg)	7.8 (0.4)	9.0 (0.3)	–1.17 (–2.21; –0.12)	0.03
Copper (mg)	2.6 (0.3)	1.3 (0.2)	1.25 (0.58; 1.92)	< 0.001
Magnesium (mg)	327 (13.6)	336 (9.7)	–9.18 (–44.7; 26.3)	0.61
Selenium (µg)	34.1 (1.9)	43.2 (1.4)	–9.15 (–14.2; –4.06)	0.001
Iodine (µg)	27.0 (3.2)	46.1 (2.3)	–19.1 (–27.5; –10.7)	< 0.001
Vitamin A (retinol, mg)	305 (136.3)	624 (97.4)	–319 (–675; 37.5)	0.08
Beta carotene (g)	2.50 (0.2)	2.03 (0.2)	0.47 (–0.14; 1.08)	0.13
Thiamin (vitamin B1,mg)	1.03 (0.1)	1.14 (0.0)	–0.11 (–0.25; 0.03)	0.12
Riboflavin (vitamin B2,mg)	1.41 (0.1)	1.30 (0.1)	0.11 (–0.15; 0.36)	0.40
Niacin (Vitamin B3, mg)	15.4 (0.9)	18.4 (0.7)	–2.97 (–5.35; –0.59)	0.02
Pyridoxine (vitamin B6, mg)	1.35 (0.1)	1.70 (0.0)	–0.36 (–0.54; –0.18)	< 0.001
Folates naturally occurring,(µg)	198 (11.6)	201 (8.3)	–2.89 (–33.2; 27.4)	0.85
Cobalamin (vitamin B12, µg)	3.18 (0.5)	4.23 (0.4)	–1.05 (–2.42; 0.33)	0.13
Vitamin C (mg)	106 (9.1)	97.8 (6.5)	8.36 (–15.4; 32.1)	0.48
Vitamin D (µg)	1.74 (0.3)	2.57 (0.2)	–0.83 (–1.49; –0.16)	0.02
Vitamin E (mg)	9.45 (0.7)	9.35 (0.5)	0.10 (–1.75; 1.95)	0.91

Data are (adjusted) means (with standard errors in parentheses) and adjusted mean differences (with 95% confidence intervals in parentheses). Adjusted values were calculated with analysis of variance adjusted for gender, age, BMI, and total caloric intake.

Supplementary Material 4 Doses of previous studies including the current study.

	Previous studies									
	Schoenthaler 1997	Schoenthaler 2000	Gesch 2002	Zaalberg 2010	Adams[a] 2011	Long 2013	Tammam 2016	Raine 2016	Rucklidge[b] 2018	**PSYVA**
**Minerals**										
**Potassium (mg)**			4	4	50	40		40	192	
**Calcium (mg)**	122	200	100	100	100	162		162	1056	
**Manganese (mg)**	3	1	3	3	3	2	2	2	7.7	**5**
**Iron (mg)**	18	9	12	12		5	12	7.2	10.8	**8**
**Zinc (mg)**	15	8	15	15	12	5	15	8.3	38.4	**7.5**
**Copper (mg)**	2	1	2	2		0.5	1	0.9	5.8	**0.5**
**Magnesium (mg)**	59	80	30	100	100	100	94	20	480	
**Molybdenum (µg)**	250	120	250	250	150	50			120	**13**
**Selenium (µg)**	100	50	50	50	22	30	55		168	**75**
**Chromium (µg)**	100	50	200	200	70	40	50		504	**13**
**Iodine (µg)**	150	75	140	140	100	100	130	160	163	**150**
**Lithium (µg)**					500					
**Sulfur (mg)**					500					
**Phosphorus (mg)**						125			672	
**Chloride (mg)**						36.3				
**Vitamins**										
**A (Retinol, mg)**	1.5	0.75	0.75	0.75	0.6	0.8	0.4	0.8	1.4	
**A (Beta carotene, mg)**		–	–	0.13						**6**
**B1 (Thiamin, mg)**	4.5	0.75	1.2	1.2	20	1.4	6	1.4	48	**15**
**B2 (Riboflavin, mg)**	5.1	0.9	1.6	1.6	20	1.75	3	1.75	14.4	**15**
**B3 (Niacinamide, mg)**	60	10	18	18	25	20	18	20	72	**20**
**B5 (Pantothenic acid, mg)**	30	5	4	4	15	7.5	6	7.5	24	**15**
**B6 (Pyridoxine, mg)**	30	1	2	2	40	2	8	2	56	**5**
**Folic acid / B11 (µg)**	400	200	400	400	100	200	400	200	640	**400**
**B12 (Canocobalamin, µg)**	18	3	3	3	500	2.5	15	2.5	720	**25**
**Biotin (µg)**		150	100	100	150	62.5	75		864	**25**
**C (Ascorbic acid, mg)**	120	40	60	60	600	100	80	100	480	**100**
**D3 (Cholecalciferol, µg)**	5	5	10	5	7.5	5	20	5	60	**25**
**E (D-alpha-tocopherol, mg)**	80	10	10	10	270	15	8	15	200	**53**
**K (µg)**	50	50				30		30	96	
**Other**										
**Inositol (mg)**	40				100				144	
**Benzoic acid (mg)**	50									
**Choline (mg)**	40				250				432	
**Fatty acids**										
**Linoleic acid (mg)**			1260			10		400		
**γ-Linolenic acid (mg)**			160	100						
**EPA (mg)**			80	400			165	200		**329**
**DHA (mg)**			44	400		673	116	300		**219**

[a] Dosage was adjusted based on baseline measured body weight up to a maximum of 100 lbs. The dosage shown is for a 60 lbs child.
[b] Target dose was based on the response rates.

Supplementary Material 5 Average costs (in €) per patient year, by randomization group.

	Supplementation		Placebo		Difference	
	Frequency	Costs	Frequency	Costs	Costs	*p* value
Supplements[a]		314		0	314	-
Distribution		79		0	79	-
**Total intervention costs**		**393**		**0**	**393**	-
Verbal incidents	3.9	197	2.3	114	83	0.16
Physical against objects	1.4	122	0.9	76	46	0.20
Physical self-harm	1.9	223	1.9	224	-1	0.99
Physical against others	15.2	2,985	16.5	3,241	-256	0.79
**Total aggression costs**	**22.4**	**3,526**	**21.5**	**3,655**	**-128**	0.90
**Total healthcare costs**		**3,919**		**3,655**	**264**	**0.81**

[a] Reported costs are for the supplements used in the study. Less expensive supplements are available for €69 per patient year

Micronutrient	n	Baseline (mean ± SE)	n	Follow-up (mean ± SE)	Test for interaction (time * group)
Vitamin A (µmol/L):					
Supplement	45	2.45 ± 0.10	33	2.68 ± 0.12	F(df. 1) = 3.68; P = 0.06
Placebo	52	2.26 ± 0.10	44	2.21 ± 0.10	
Vitamin B1 (nmol/L):					
Supplement	44	13.2 ± 1.7	32	19.3 ± 2.0	F(df. 1) = 2.74; P = 0.10
Placebo	49	13.2 ± 1.6	41	14.1 ± 1.7	
Vitamin B6 (nmol/L):					
Supplement	45	86.7 ± 9.89	33	129.0 ± 11.5	F(df. 1) = 7.97; P = 0.005
Placebo	51	59.6 ± 9.29	43	51.8 ± 10.1	
Folic Acid (nmol/L):					
Supplement	44	10.4 ± 1.1	33	26.8 ± 1.2	F(df. 1) = 53.40; P < 0.001
Placebo	52	10.3 ± 1.0	44	10.5 ± 1.1	
Vitamin B12 (pmol/L):					
Supplement	44	439 ± 28.2	33	547 ± 30.9	F(df. 1) = 4.22; P = 0.04
Placebo	52	367 ± 26.0	44	397 ± 27.4	
Vitamin D3 (nmol/L):					
Supplement	44	98.5 ± 6.0	33	112.0 ± 6.5	F(df. 1) = 2.86; P = 0.09
Placebo	52	88.0 ± 5.6	44	89.7 ± 5.8	
Vitamin E (µmol/L):					
Supplement	45	27.9 ± 1.2	33	32.5 ± 1.3	F(df. 1) = 5.17; P = 0.02
Placebo	52	27.0 ± 1.1	44	27.5 ± 1.2	
Iron (µmol/L):					
Supplement	44	16.2 ± 0.9	33	17.5 ± 1.0	F(df. 1) = 2.34; P = 0.13
Placebo	52	14.8 ± 0.8	44	13.9 ± 0.9	
EPA (mass%):					
Supplement	45	0.80 ± 0.08	33	1.64 ± 0.09	F(df. 1) = 20.85; P < 0.001
Placebo	52	0.65 ± 0.07	44	0.92 ± 0.07	
DHA (mass%):					
Supplement	45	2.85 ± 0.15	33	5.20 ± 0.17	F(df. 1) = 24.09; P < 0.001
Placebo	52	2.42 ± 0.14	44	3.56 ± 0.15	

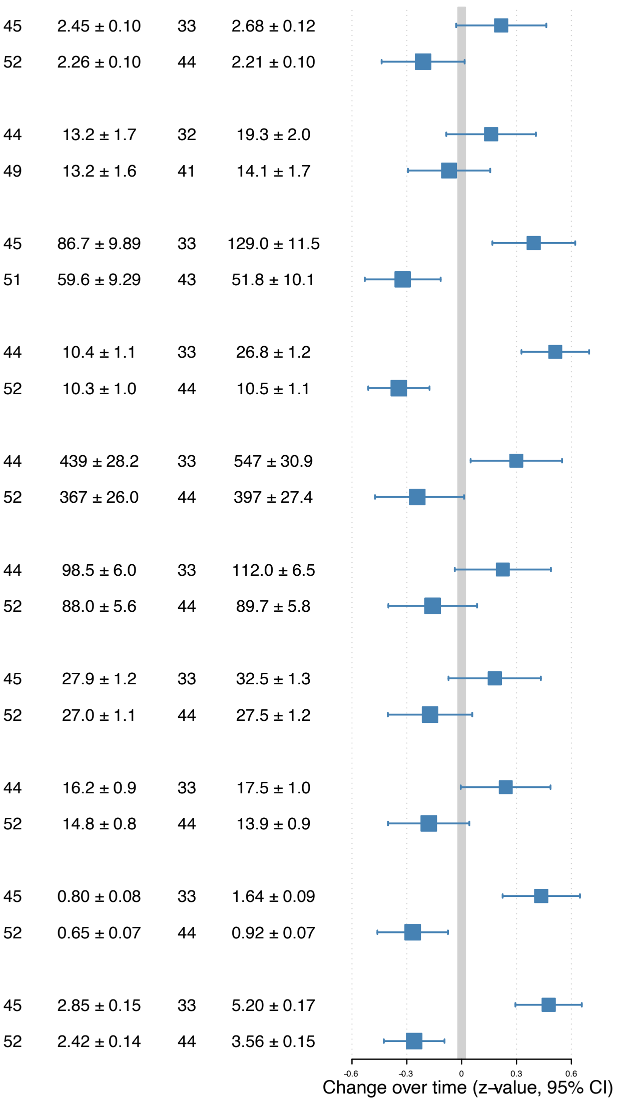

Supplementary Material 6 Changes in micronutrient levels between supplementation and placebo.

Note. Reference values as used by the LUMC: Vitamin A=0.7 – 2.6; Vitamin B1=78 – 143; Vitamin B6=51 – 183; Folic acid=8.8 – 60.8; Vitamin B12=150 – 700; Vitamin D=50 – 250; Vitamin E=16.0 – 41.0; Iron=♂ 14 – 35; Iron=♀ 10 – 25.

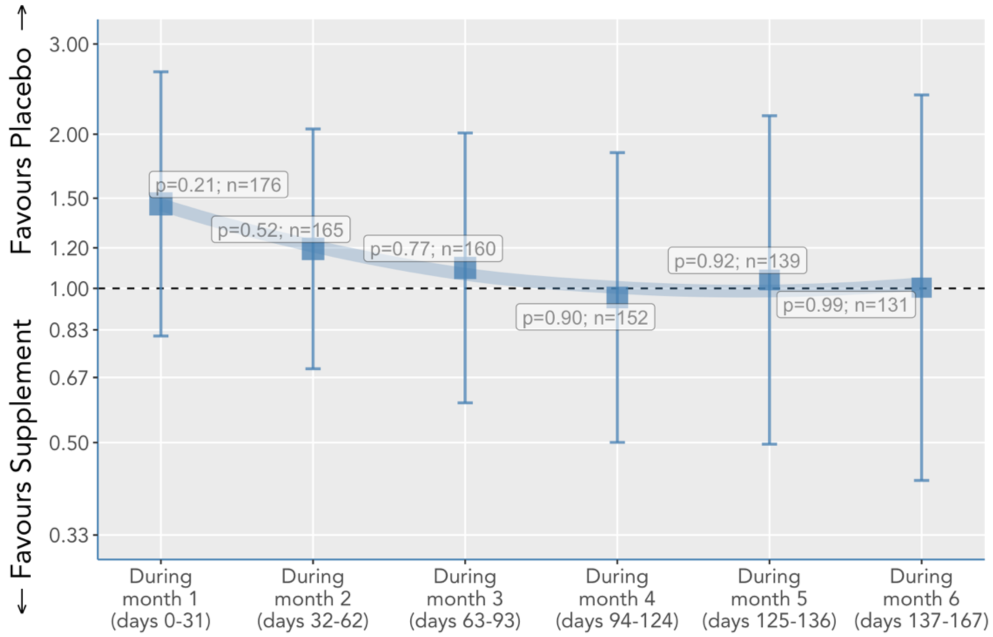

Supplementary Material 7 Effect on aggressive incidents over time during 6 months of supplementation.

Subgroup	n	Rate Ratio (95% CI)	Mean no of inc/mo Supplement	Mean no of inc/mo Placebo	P for interaction
Main effect	176	1.08 (1.74-0.67)	1.03 (0.74-1.37)	0.90 (0.65-1.19)	
Gender:					0.757
Male	113	0.96 (0.53-1.73)	0.71 (0.45-1.01)	0.70 (0.46-0.98)	
Female	63	1.11 (0.54-2.26)	1.69 (1.01-2.60)	1.36 (0.76-2.15)	
Age:					0.120
<50 y	82	1.66 (0.85-3.22)	1.03 (0.58-1.61)	0.73 (0.45-1.06)	
>= 50 y	94	0.79 (0.41-1.52)	1.03 (0.66-1.48)	1.07 (0.66-1.58)	
Ward:					0.678
Closed	111	1.10 (0.63-1.92)	1.28 (0.84-1.83)	1.10 (0.73-1.55)	
Open	65	0.90 (0.41-1.98)	0.64 (0.37-0.97)	0.61 (0.33-0.96)	
Maried:					0.836
Never	132	1.03 (0.58-1.83)	0.83 (0.53-1.18)	0.87 (0.60-1.18)	
Ever	35	1.17 (0.45-3.06)	1.89 (1.03-3.11)	0.90 (0.25-1.89)	
BMI:					0.616
<30 kg/m2	55	1.09 (0.48-2.48)	1.22 (0.65-1.98)	0.95 (0.49-1.56)	
>=30 kg/m2	100	0.83 (0.44-1.58)	0.84 (0.53-1.23)	0.93 (0.60-1.33)	
Ancestry:					0.312
European	141	1.13 (0.68-1.88)	1.22 (0.85-1.65)	0.99 (0.68-1.35)	
Non-European	35	0.61 (0.20-1.85)	0.37 (0.08-0.75)	0.61 (0.24-1.08)	
Current smoker:					0.533
No	125	1.24 (0.72-2.12)	0.92 (0.60-1.30)	0.83 (0.57-1.12)	
Yes	47	0.89 (0.32-2.44)	1.29 (0.67-2.14)	1.01 (0.40-1.88)	
Current alcohol:					0.439
No	142	1.22 (0.72-2.09)	1.11 (0.75-1.55)	0.85 (0.59-1.17)	
Yes	16	0.63 (0.15-2.63)	0.56 (0.05-1.32)	1.10 (0.37-2.20)	
Ever recreational drugs:					0.542
No	76	1.41 (0.71-2.79)	1.35 (0.88-1.93)	0.81 (0.45-1.26)	
Yes	94	1.05 (0.56-1.99)	0.71 (0.39-1.10)	0.86 (0.58-1.20)	
Diagnosis:					0.880
Psychotic disorder	107	1.13 (0.61-2.09)	0.88 (0.54-1.29)	0.84 (0.56-1.18)	
Other	68	1.05 (0.51-2.14)	1.34 (0.81-2.02)	0.99 (0.54-1.58)	
Antipsychotics:					0.004
No	14	0.08 (0.01-0.59)	0.30 (0.08-0.57)	1.61 (0.33-4.10)	
Yes	145	1.45 (0.88-2.39)	1.18 (0.83-1.59)	0.84 (0.59-1.13)	
Classic antipsychotics:					0.581
No	85	0.94 (0.47-1.90)	0.96 (0.56-1.47)	0.85 (0.51-1.26)	
Yes	74	1.24 (0.62-2.47)	1.28 (0.79-1.91)	1.03 (0.60-1.58)	
Atypical antipsychotics:					0.570
No	46	0.99 (0.45-2.21)	1.73 (0.92-2.89)	1.30 (0.64-2.22)	
Yes	113	1.36 (0.75-2.47)	0.95 (0.62-1.34)	0.76 (0.51-1.06)	
Antidepressants:					0.335
No	98	0.90 (0.46-1.77)	0.86 (0.52-1.29)	0.87 (0.54-1.27)	
Yes	61	1.46 (0.73-2.91)	1.62 (1.00-2.44)	0.99 (0.55-1.57)	

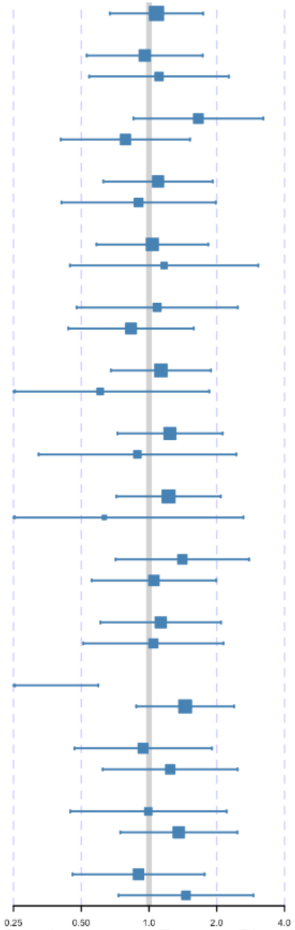

Supplementary Material 8 Subgroup analyses of the effect of supplementation on aggressive incidents according to demographic and clinical data.

Supplementary Material 9 Adverse effects in both groups.

**Side effect**	**Placebo**	**Supplements**	***p* value**
Any side effect, no (%)	15 (20.5%)	17 (23.0%)	0.72
Nauseous, no (%)	4 (5.6%)	2 (2.7%)	0.39
Diarrhea, no (%)	3 (4.2%)	6 (8.2%)	0.31
Burping, no (%)	2 (2.8%)	8 (11.1%)	0.049
Tiredness, no (%)	3 (4.2%)	4 (5.5%)	0.71
Rash, no (%)	0 (0.0%)	4 (5.5%)	0.04
Headache, no (%)	3 (4.2%)	3 (4.1%)	0.99
Sleep problems, no (%)	2 (2.8%)	3 (4.1%)	0.66
Weight gain, no (%)	2 (2.8%)	2 (2.7%)	0.99
Weight loss, no (%)	3 (4.2%)	1 (1.4%)	0.30
Other, no (%)	5 (6.9%)	3 (4.1%)	0.44
