# Supplementary material for: Effects of multivitamin, mineral and n-3 polyunsaturated fatty acid supplementation on aggression among long-stay psychiatric in-patients: randomised clinical trial
Source: BJPsych Open. 2022 Feb 3;8(2):e42. doi: 10.1192/bjo.2022.8 (PMC8867900; doi:10.1192/bjo.2022.8)
Supplement: Supplementary file 1 [file bjosup.zip › S2056472422000084sup003.pdf]

Diet and Aggression

Multivitamin, mineral and n-3 PUFA supplementation to reduce aggression among chronically admitted psychiatric patients: a randomized clinical trial

TABLE OF CONTENTS

1. INTRODUCTION AND RATIONALE ... 9
2. OBJECTIVES ... 11
3. STUDY DESIGN ... 12
4. STUDY POPULATION ... 14
   4.1 Population (base) ... 14
   4.2 Inclusion criteria ... 14
   4.3 Exclusion criteria ... 14
   4.4 Sample size calculation ... 15
5. TREATMENT OF SUBJECTS ... 15
   5.1 Investigational product/treatment ... 15
6. INVESTIGATIONAL PRODUCT ... 15
   6.1 Name and description of investigational products ... 15
   6.2 Summary of findings from non-clinical and clinical studies ... 16
   6.3 Summary of known and potential risks and benefits ... 19
   6.4 Description and justification of route of administration and dosage ... 19
   6.5 Dosages, dosage modifications and method of administration ... 19
   6.6 Preparation and labelling of Investigational Medicinal Product ... 20
   6.7 Drug accountability ... 20
7. METHODS ... 20
   7.1 Study parameters/endpoints ... 20
      7.1.1 Main study parameter/endpoint ... 20
      7.1.2 Secondary study parameters/endpoints (if applicable) ... 20
      7.1.3 Other study parameters (if applicable) ... 21
   7.2 Randomisation, blinding and treatment allocation ... 21
   7.3 Study procedures ... 22
      7.3.1 Enrolment and short qualitative study ... 22
      7.3.2 Randomisation procedure, baseline assessment and intervention ... 22
      7.3.3 Follow-up assessments ... 23
      7.3.4 Data collection ... 23
   7.4 Withdrawal of individual subjects ... 24
   7.5 Replacement of individual subjects after withdrawal ... 24
   7.6 Follow-up of subjects withdrawn from treatment ... 25
   7.7 Premature termination of the study ... 25
8. SAFETY REPORTING ... 25
   8.1 Section 10 WMO event ... 25
   8.2 AEs, SAEs and SUSARs ... 25
      8.2.1 Adverse events (AEs) ... 25
      8.2.2 Serious adverse events (SAEs) ... 25
   8.3 Follow-up of adverse events ... 26
9. STATISTICAL ANALYSIS ... 26
   9.1 Primary study parameter ... 26

9.2 Secondary study parameters ... 27
10. ETHICAL CONSIDERATIONS ... 28
10.1 Regulation statement ... 28
10.2 Recruitment and consent ... 28
10.3 Objection by minors or incapacitated subjects (if applicable) ... 28
10.4 Benefits and risks assessment, group relatedness ... 29
Benefits ... 29
10.5 Compensation for injury ... 30
10.6 Incentives ... 30
11. ADMINISTRATIVE ASPECTS, MONITORING AND PUBLICATION ... 30
11.1 Handling and storage of data and documents ... 30
11.2 Amendments and add-on studies ... 31
11.3 Annual progress report ... 31
11.4 End of study report ... 31
11.5 Public disclosure and publication policy ... 32
12. STRUCTURED RISK ANALYSIS ... 32
12.1 Potential issues of concern ... 32
13. Reference List ... 35

LIST OF ABBREVIATIONS AND RELEVANT DEFINITIONS

**ABR**	ABR form, General Assessment and Registration form, is the application form that is required for submission to the accredited Ethics Committee (In Dutch, ABR = *Algemene Beoordeling en Registratie*)
**ADH**	Recommended Daily Dosage (in Dutch: *Aanbevolen dagelijkse hoeveelheid*)
**AE**	Adverse Event
**ALA**	Alpha-Linolenic Acid
**AR**	Adverse Reaction
**Atrium Innovations Inc.**	Atrium Innovations Inc. develops, manufactures, and markets products for the cosmetics, pharmaceutical, chemical, and nutrition industries and is owner of the Orthica brand.
**AVL-AV**	*Aangepaste Versie van de Agressie Vragenlijst* [1], a 12-item self-report questionnaire on feelings of aggression and irritability.
**BMI**	Body Mass Index
**BZK**	Ministry of National and Kingdom Affairs (in Dutch: *Ministerie van Binnenlandse Zaken en Koninkrijksrelaties*)
**CCMO**	Central Committee on Research Involving Human Subjects (in Dutch: *Centrale Commissie Mensgebonden Onderzoek*)
**CI**	Confidence Interval
**CRF**	Case Report Form
**CV**	Curriculum Vitae
**DHA**	Docosahexaenic acid
**EDTA**	Iron Chelator in blood collection tubes
**EFSA**	European Food Safety Administration
**EMP+**	EMPower Plus
**EPA**	Eicosapentaenic Acid
**EU**	European Union
**FAO**	Food and Agriculture Organisation
**FFQ**	Food Frequency Questionnaire
**GABA**	γ-amino butyric acid
**GCP**	Good Clinical Practice
**GGZ**	Mental Healthcare (in Dutch: *Geestelijke Gezondheidszorg*)
**IB**	Investigator's Brochure
**IC**	Informed Consent

**ITT**	Intention To Treat
**LUMC**	Leiden University Medical Centre
**METC**	Medical Research Ethics Committee (MREC) (in Dutch: *Medisch Ethische Toetsings Commissie*)
**n-3FA**	n-3 Fatty Acids, i.e. omega-3 fatty acids
**n-9FA**	Neutral oils that will be used in placebo supplements
**PINUP**	PrIson NUtrition Project
**PP**	Per Protocol
**PUFA**	Polyunsaturated Fatty Acids
**RCT**	Randomised Controlled Trial
**RDA**	Recommended Daily Amount
**(S)AE**	(Serious) Adverse Event
**SD**	Standard Deviation
**SDAS**	Social Dysfunction and Aggression Scale [2], an 11-item observer rated questionnaire measuring aggression and hostility
**SOAS-R**	Staff Observation Aggression Scale- Revised version [3], a standardised registration form for aggressive incidents that can be filled in by nursing staff
**SPC**	Summary of Product Characteristics (in Dutch: *Officiële productinfomatie IB1-tekst*)
**Sponsor**	The sponsor is the party that commissions the organisation or performance of the research, for example a pharmaceutical company, academic hospital, scientific organisation or investigator. A party that provides funding for a study but does not commission it is not regarded as the sponsor, but referred to as a subsidising party
**SSRI**	Selective Serotonin Reuptake Inhibitor
**SUSAR**	Suspected Unexpected Serious Adverse Reaction
**VWS**	Ministry of Health, Wellbeing and Sports (in Dutch: *Ministerie van Volksgezondheid, Welzijn en Sport*)
**vCPRS**	*Verkorte* Comprehensive Psychopathological Rating Scale [4], a 25-item observer rated questionnaire measuring affective symptoms
**VJ**	Ministry of Safety and Justice (in Dutch: *Ministerie van Veiligheid en Justitie*)
**WBP**	Personal Data Protection Act (in Dutch: *Wet Bescherming Persoonsgevens*)

**WGBO**	Medical Treatment Act (in Dutch: *Wet op de Geneeskundige Behandelovereenkomst*)
**WHOQL-bref**	World Health Organization Quality of Life [5], a 26-item self-report questionnaire measuring quality of life
**WMO**	Medical Research Involving Human Subjects Act (in Dutch: *Wet Medisch-wetenschappelijk Onderzoek met mensen*)
**ZonMW**	Main subsidising party for this study, ZonMW is the Dutch organisation for health research and healthcare innovation

SUMMARY

Rationale: Aggressive incidents are highly prevalent among chronic psychiatric inpatients. Previous studies have demonstrated the potential of multivitamin-, mineral-, and n-3 fatty acids (n-3FA) supplementation to reduce aggression in adolescent and forensic populations.

Objective: To test the hypothesis that multivitamin-, mineral-, and n-3FA supplementation reduces aggression among chronic psychiatric inpatients.

Study design: Pragmatic, randomised, double blind, placebo controlled, multicentre intervention study.

Study population: Psychiatric inpatients residing in long-stay psychiatric wards.

Intervention: During 6 months one group receives 3 supplements daily: 2 Orthica Multi Energie (vitamins [B1, B2, B3, B5, B6, B11, B12, C, D, E, Beta Carotene] and minerals [Iodine, Copper, Selenium, Iron, Zinc, Potassium, Chrome, Manganese, Molybdenum]) and 1 Orthica Fish EPA Mini (n-3FA: eicosapentaenic acid [EPA] and docosahexaenic acid [DHA]) the other group receives 3 placebo capsules.

Main study parameters/endpoints: The main study parameter is the number of aggressive incidents from baseline (t0) to endpoint (six months post baseline, t3), as measured using the Dutch version of the 'Staff Observation Aggression Scale- Revised version' (SOAS-R) [3].

Nature and extent of the burden and risks associated with participation, benefit and group relatedness: Patients who wish to participate enter a 2-week run-in phase in which they take 3 placebo capsules daily. After positive evaluation of this run-in phase patients are randomised to active or control condition. Participants will then start the daily use of 3 supplement capsules or 3 placebo capsules, which continues for 6 months. At 3 points (t0, 2 months post baseline [t2] and t3) 3 questionnaires will be administered: the *Aangepaste Versie van de Agressievragenlijst* (AVL-AV, [1]), a 12 item self-report questionnaire about feelings of aggression; the World Health Organization Quality of Life Questionnaire (WHO-QL-bref [5]), a 26-item observer rated quality of life instrument; and the *verkorte* Comprehensive Psychopathological Rating Scale (vCPRS [4]), a 25-item observer rated instrument on affective symptoms. Also, at t0 and t3, blood samples (50cc) will be taken to determine nutrient status. All data collection will take place at the department where the patient resides. Aggression incidents will be registered by nursing staff in the department using the SOAS-R [3]. As registration of severe incidents is already part of standard care, this registration will not burden the patient. Also, at 4 time points (t0, 2 weeks post baseline [t1], t2 and t3), nursing staff will fill in the Social Dysfunction Aggression Scale (SDAS [2]), measuring observed levels of aggression and social dysfunction.

The risks of participating in this study are minimal, the risk of venapuncture is that of a small local hematoma, feeling light headed, fainting or local infection. The use of the supplements Orthica Multi Energie and Orthica Fish EPA Mini has not been associated with any significant

risks. Burden associated with participation is also minimal with data collection involving 3 questionnaire administrations and two blood sample collections. Participants will be compensated for their time. Potential benefits of participating in this study are a direct and indirect increase in quality of life through improved nutrition as well as reduction of aggressive incidents. As this is a pragmatic trial in which the main objective is to determine whether the use of supplements has the potential to reduce the number of aggressive incidents in chronic psychiatric inpatients, it is imperative to involve these patients.

1. INTRODUCTION AND RATIONALE

Aggressive incidents frequently occur among long-term psychiatric inpatients (n=12.201 in The Netherlands [6]). Incidents, such as verbal aggression towards persons, aggression towards objects, threats, non-compliance with hospital rules, disinhibited (sexual) behaviour, fighting, assault on patients or staff, self-harm and suicide attempt; follow from a wide range of causes such as psychosocial stressors, patient history, psychopathology, resistance to treatment, and being institutionalized. The magnitude of the aggression problem becomes evident in a review of 122 studies conducted in various psychiatric settings (5 of which in The Netherlands), showing that severe aggressive incidents occurred on average 5.8 times per 100 occupied bed days, amounting to 21 (standard deviation [SD]=42) serious events per bed per year [7]. The large SD indicates extreme variability across studies. As the numbers reported in this review were based on psychiatric hospitals in general and not on long-stay units in particular, we conducted a pilot study in 3 chronic inpatient wards (in Oegstgeest, The Hague, and Amsterdam). Between February and March 2014, we recorded prevalence of aggression and its financial consequences. Results demonstrated an incidence rate of 90 aggression incidents per patient per year. This number can be divided in 19 *major* incidents (severe threats, fighting, assault on patients or staff, self-harm, suicide attempt), and 71 *minor* incidents (verbal aggression, threats, non-compliance with hospital rules, aggression towards objects, disinhibited [sexual] behaviour). This number is higher than what was previously reported, which may be due to the specific focus on long-stay units, as well as the inclusion of less severe (verbal) aggression incidents.

Aggression has serious consequences; incidents can have physical consequences, may cause stress, and can be traumatic for patients as well as staff [8 7 9]. Consequences of aggression can also be expressed in terms of financial costs. Finally, aggression is one of the reasons long-term psychiatric inpatients remain admitted involuntarily [10]. Policymakers prioritize aggression reduction in (mental) health care. Containment of aggression and its consequences is achieved through (coerced) medication, observation, show of force, restraint, seclusion, time-out, and security policies like locking ward doors, but also through aggression-anticipation and environment- and client-focused approaches [7]. However, further reduction of aggressive incidents remains necessary as becomes evident from initiatives like the petition "*handen af van ggz verpleegkundigen*" (www.handenafvanggzverpleegkundigen.nl), and the action plan against aggression in care (http://www.rijksoverheid.nl/nieuws/2012/03/22/ministers-presenteren-gezamenlijk-actieplan-tegen-agressie-in-de-zorg%5B2%5D.html), an initiative of the ministries of *Volksgezondheid, Welzijn en Sport* (VWS), *Binnenlandse Zaken en Koninkrijksrelaties* (BZK), *Veligheid &*

Justitie (VJ), and social parties. The need for aggression reduction is also felt strongly among nursing staff, as is reflected in responses to a short anonymous online survey we conducted among psychiatric nurses (n=19), who unanimously endorsed the need for new methods for reducing aggression.

A number of randomised controlled trials (RCTs) demonstrated anti-aggressive effects of multi-ingredient formulae, including multiple vitamins, minerals, and n-3FA (i.e., omega-3 fatty acids), see figure 1. An RCT of vitamins and minerals in 62 juvenile delinquents showed a 28% (95% confidence interval [CI]: 18-45%) decrease in violent and nonviolent offences over a 13-week period [11]. A subsequent RCT of vitamins and minerals in 80 frequently disciplined schoolchildren showed a 47% (95%CI: 29-65%) reduction in the number of violent and nonviolent delinquent acts during a 4-month period [12]. An RCT of vitamins, minerals, and n-3FA in 231 prisoners showed a 26% (95%CI: 8-44%) reduction in the number of offences and antisocial behaviour during a 2-39 week period [13]. Another vitamin, mineral, and n-3FA-RCT in 221 young adult prisoners demonstrated an incidence rate ratio of 0.60 (95%CI: 0.37-0.96) during a 1-3 month period [14]. Finally, a pilot study with 12 treatment resistant schizophrenia patients demonstrated reduced agitation and psychopathology and increased functioning upon n-3FA supplementation [15].

Chronic psychiatric inpatients are known to have poor nutritional status [16 17]. This is the result of energy-dense and nutrient-poor diets, low appetite, and insufficient outdoor activities but also of the detrimental effect of psychotropics on appetite and gastrointestinal function, and possible interactions with food and nutrients [18]. Based on the high prevalence of aggression and the poor nutritional status of this patient group, we hypothesize that the aggression reducing effect of vitamin-, micronutrient-, and n-3FA-supplementation in this group may be substantial. Providing nutritional supplements to this patient group may result in the reduction of aggression, a decrease of costs related to aggression, and an increase in patients' quality of life. We therefore propose a pragmatic randomised double-blind placebo controlled multicentre intervention trial.

Figure 1. Overview of studies of anti-aggressive effects of multi-ingredient formulae, including multiple vitamins, minerals, and n-3 fatty acids (n-3FA) (i.e., omega-3 fatty acids)

Author	year	n	all pl/act	completed	duration	age	subjects	
Schoenthaler	1997	71	66	30/32	3 mnt	13-17	incarcerated juveniles	(P=0.005)
Schoenthaler	2000	468	234/234	192/196	4 mnt	6-12	disciplined schoolchildren	(P=0.04)
Gesch	2002	231	115/116	90/82	2-39 wk	18-23	young adult prisoners	(*P*=0.03)
Zaalberg	2010	326	NA	106/115	1-3 mnt	18-25	young adult prisoners	(*P*=0.02, one-tailed)

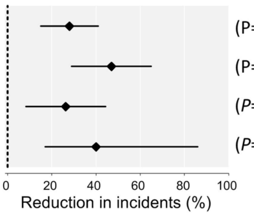

Act denotes active treatment; mnt, month; NA, not available; pl, placebo.

2. OBJECTIVES

Primary objective

The primary objective of this study will be to test the hypothesis that multivitamin-, mineral- and n-3FA supplementation is effective in aggression reduction in chronic psychiatric inpatients. The main research question is whether the number of aggressive incidents decreases after supplementation.

Secondary objectives

Secondary objectives are:

- the identification of possible barriers in the acceptation of nutritional supplements by chronic psychiatric inpatients
- determining cost-effectiveness of supplementation through reduction of costs of time spent by staff members on aggression incidents and additional costs of incidents
- determining compliance through analysing nutrient status in blood samples
- determining effect of supplementation on patients' self-report aggressive and hostile feelings (AVL-AV) [1]
- determining effect of supplementation on patients' observed aggressive and hostile feelings (SDAS) [2]
- determining effect of supplementation on patients' observed affective symptoms (vCPRS) [4]
- determining effect of supplementation on patients' self-report quality of life (WHO-QL-Bref) [5]

3. STUDY DESIGN

The Diet and Aggression study is a pragmatic randomised double-blind placebo controlled multicentre intervention trial. The main applicant for the Diet and Aggression study is the department of psychiatry of the Leiden University Medical Centre (LUMC). The study will take place in approximately 15 institutions for long-term psychiatric inpatient care in The Netherlands. At present 7 institutions (Parnassia, The Hague; GGZ Rivierduinen, Oegstgeest; GGZ Delfland, Delft, Schiedam; GGZ Centraal, Ermelo; GGZ Eindhoven, Eindhoven; Fivoor, Den Dolder, The Hague, PZ Bethaniënhuis, Zoersel, Belgium; Multiversum, Mortsel, Belgium) are included in our protocol and are recruiting participants. As the washout period of multivitamin, mineral and n-3FA supplementation regarding aggression differs across supplements or is unknown, we chose a parallel design. Patients are randomised to placebo or active supplements (see section 6 INVESTIGATIONAL PRODUCT and supplementary Material 2).

Study start-up and inclusion will take approximately 24 months. During this period, eligible patients will be identified. Study objectives will be communicated to patients and relatives through the patient information folder, accompanying flyers, and in verbal communication with the on-site principal investigator. Patients or their representatives will provide informed consent. Nurses will be trained on registering aggression using the SOAS-R. To maximize compliance in this patient group in which medication resistance is common [19], a short survey aimed at addressing barriers towards the use of supplements has been developed. To this end, barriers/ facilitators of supplement acceptation have been identified in a series of semi-structured interviews with approximately 5 to 10 patients. When there was no data saturation after 5-10 interviews, we continued until three consecutive interviews emerged without new ideas (stopping criterion) [16]. In these semi-structured interviews, the interviewer explored the barriers and facilitators at different levels (the innovation [i.e. nutritional supplementation], the professional, the organizational and external context [political, social and economic factors]), using the framework that has been developed by Grol [17]. The interviews have been audio-taped and qualitatively analysed. Based on the results of the interviews, a survey to quantify the barriers and facilitators has been prepared that is used for evaluation of supplement use after the two-week run-in phase.

The intervention/ assessment phase will take place over a period of approximately 12 months and is shown in figure 2. Patients will start with a short run-in phase of two weeks during which they will take three placebo capsules on a daily basis. After this period, supplement use will be evaluated using the short survey aimed at barriers towards supplement acceptation (t0), after positive evaluation, patients will be randomised to active or control condition. Patients will then start taking three supplement or placebo capsules on a daily basis over a period of six months. During the intervention phase, detailed registration of

aggression incidents will be carried out continuously by nursing staff using the SOAS-R [3]. In addition data collection with the AVL-AV [1] (self-report), the SDAS [2] (observer rated, filled in by nursing staff), the vCPRS [4] (observer rated) and the WHOQL-Bref [5] (self-report) will take place at baseline (t0), at two-month follow up (t2), and at 6-month follow-up (t3). Also at baseline, patient background information will be collected (BMI, blood pressure, medication and social demographic information). The SDAS [2] (observer rated) will also be filled in by nurses at two weeks after start of the intervention (t1). Finally, blood samples will be collected to determine nutritional status (a selection of serum levels of key vitamins, minerals and n-3FA) and monitor compliance at t0 and t3.
The analysis/ reporting phase will take an additional 12 months. During this period, primary analyses, comparing numbers of aggression incidents between experimental and control condition, and secondary analyses on costs of care, aggression scores, affective symptoms, and quality of life, will be conducted. Results will be reported at national and international conferences, and will be communicated within institutions. The total duration of the Diet and Aggression study will be 48 months from the start of the first inclusion onwards.

Figure 2. Flowchart of the intervention phase of the Diet and Aggression study.

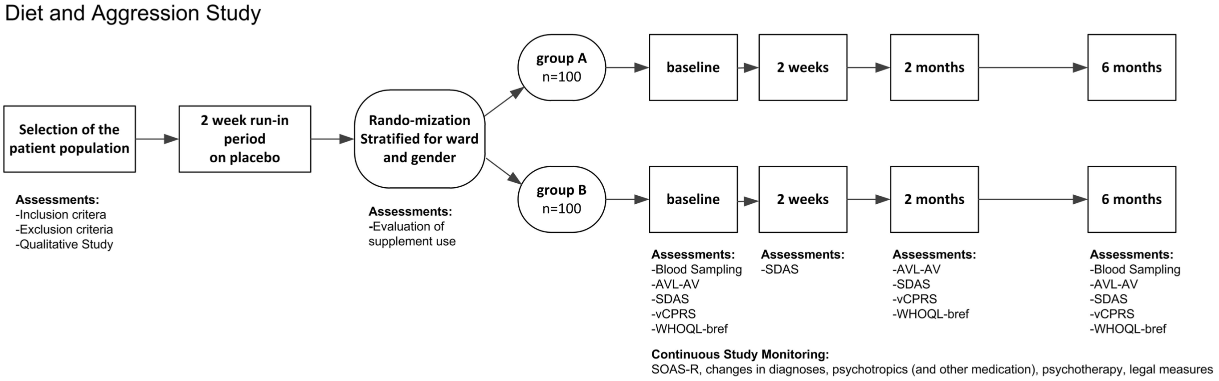

AVL-AV denotes Aangepaste Versie van de Agressie Vragenlijst [1]; SOAS-R denotes Staff Observation Aggression Scale-Revised version [3]; SDAS denotes Social Dysfunction and Aggression Scale [2]; vCPRS denotes Comprehensive Psychopathogical Rating Scale [4]; WHOQL-bref denotes World Health Organization Quality of Life [5].

4. STUDY POPULATION

4.1 Population (base)

The Diet and Aggression study is a pragmatic trial; the main research question is whether nutritional supplements are effective in reducing aggression among patients residing in long-term inpatient care facilities. Therefore, the source population consists of all patient's residing at facilities for long-term psychiatric inpatient care. Facilities for long-term psychiatric care are defined as facilities where adult patients reside for an average period of one year or more. In The Netherlands, this population is estimated to consist of 12.201 patients [6]. We aim to include a total of 200 patients. The average unit for long-term psychiatric care consists of approximately 20 to 30 beds. Although almost all patients will meet inclusion criteria, we expect approximately 50% of patients will agree to take part in the study and have a positive evaluation of the run-in phase. Therefore, involvement of 15 clinics will ensure the inclusion of 200 patients. The number of patients included per participating facility will reflect the distribution of patients across the various participating institutions in The Netherlands.

4.2 Inclusion criteria

In order to be eligible to participate in this study, a subject must meet all of the following criteria:

- age 18 or older
- residing at a facility for long-term psychiatric inpatient care

4.3 Exclusion criteria

A potential subject who meets any of the following criteria will be excluded from participation in this study:

- pregnancy
- breastfeeding
- known contra-indication for treatment with the supplements used in this study
- expected discharge or transfer to a non-participating centre within the next eight weeks
- current use or use in the past eight weeks of nutritional supplements and unwillingness to quit, with the exception of vitamin B1 (thiamine) and vitamin D
- Contra-indication for the consumption of pork-gelatine
- Failure to complete the two-week run-in phase (see chapter 3 and 7.3.2)

4.4 Sample size calculation

As described in the manuscript.

5. TREATMENT OF SUBJECTS

5.1 Investigational product/treatment

During the two-week run-in phase participants will receive three placebo capsules consisting of neutral oils (n-9FA) daily.
During the six-month intervention, one group will receive three daily supplements (see chapter 6 investigational product):

- 2 Orthica Multi Energie, containing vitamins (B1, B2, B3, B5, B6, B11, B12, C, D, E, Beta Carotene) and minerals (Iodine, Copper, Selenium, Iron, Zinc, Chrome, Manganese, Molybdenum).
- 1 Orthica Fish EPA Mini, containing n-3FA (EPA and DHA).

Both supplements are soft gel capsules and are available to the general public without prescription. Both supplements can be used as an addition to the existing diet. Patients can continue their normal dietary pattern and use of medication. Dosages are described in more detail in section 6 and Supplementary Material 2 and correspond to the effective dosages in previous trials [11] [12] [13] [14], except for a higher dose of B-vitamins in our study (see Supplementary Material 2). The other group will receive three placebo capsules daily. Both supplements and placebos will be distributed by nursing staff, together with regular medication, or through patients' Baxters, which are filled by the local pharmacist.

6. INVESTIGATIONAL PRODUCT

6.1 Name and description of investigational products

The investigational products in this study are Orthica Fish EPA Mini and Orthica Multi Energie. Orthica Fish EPA Mini is an over-the-counter food supplement, containing n-3FA (EPA and DHA). Orthica Multi Energie is an over-the-counter food supplement containing vitamins (B1, B2, B3, B5, B6, B11, B12, C, D, E, Beta Carotene) and minerals (Iodine, Copper, Selenium, Iron, Zinc, Chrome, Manganese, Molybdenum). Both supplements are soft gel capsules and can be used as an addition to the existing diet. Patients can continue their normal dietary pattern and use of medication. Dosages are described in more detail in the SPC and Table 1 and correspond to the effective dosages in previous trials [11] [12] [13] [14], except for a higher dose of B-vitamins in our study (see Table 1). The placebo group will receive capsules with neutral oils (n-9 FA) that are produced by Atrium Innovations Inc.

(producer of Orthica products), and are similar in look, taste, feel and smell to Orthica Fish EPA Mini and Orthica Multi Energie.

6.2 Summary of findings from non-clinical and clinical studies

The studies that form the basis for our proposal (Schoenthaler 1997} [12] Gesch} [14] have used multi-ingredient formula (e.g., the Dutch study by Zaalberg et al. used a 26-ingredient formula [14]). Formulae included a mix of vitamins, micronutrients (e.g. the trace elements zinc, iron) and the n-3FA EPA and DHA. The precise mechanisms of action are not well understood, and may in part be due to underlying dietary inadequacy in prisoners (that may similarly exist in in psychiatric inpatients). It is unclear whether the responses found were due to the vitamins, minerals or fatty acids, although it may well be the combination. Previous preclinical and epidemiological research suggested that several micronutrients may affect the risk of aggressive and violent behavior [21]. The central nervous system is such a complex organ that small deficiencies may have a cascade of adverse effects on its delicate and highly active metabolism [20]. It is speculated that mineral, vitamins and fatty acids play a role in regulating monoaminegic neurotransmitters and neurohormones.
There is evidence that poor quality nutrition and malnutrition impairs brain function. Malnutrition during gestation at the end of World War II in The Netherlands was associated with an increased risk of antisocial personality disorder [22] which was replicated in Mauritius[23]. Management of aggression and violent incidents of psychiatric inpatients now focusses on prophylactic measures and emergency treatment of acute violent behavior. Seclusion of patients may be a safe and necessary intervention. Pharmacological treatments may involve intramuscular administration of sedative and antipsychotic medication (e.g., lorazepam, haloperidol, and clozapine). Psychiatric inpatients are known to have poor nutritional status [24] [25], partly resulting from energy-dense and nutrient-poor diets, and low appetite and outdoor activities (e.g. vitamin D). Moreover, psychotropics affect appetite and gastrointestinal function, and may interact with food and nutrients in detrimental ways [18].

B Vitamins

Vitamin B1 (or thiamine) is well known in psychiatry, because of the risk of deficiency with alcoholism and eating disorders, resulting in Wernicke encephalopathy and Korsakoff's psychosis, with memory impairment, confabulation, lack of coordination, paralysis, and nystagmus. Deficiency could lead to blood-brain barrier dysfunction. Vitamin B2 (or riboflavin) functions as a cofactor in glycolysis and oxidative pathways and is essential for monoamine synthesis of neurotransmitters. Excessive alcohol intake is also associated with this deficiency, resulting in fatigue. Vitamin B6, vitamin B12 and folate play crucial roles in

metabolic pathways, including the formation of neurotransmitters epinephrine, norepinephrine, serotonin, and γ-amino butyric acid (GABA) [21]. Serotonin plays a key role in impulsive aggression and violence, as found in many studies [26 27]. Deprivation of the amino acid tryptophan, the dietary precursor of serotonin, induces aggressiveness [28], and the selective serotonin reuptake inhibitor (SSRI) fluoxetine had anti-aggressive effects in subjects with personality disorders [29 30]. Vitamin B6 (i.e., pyridoxine, pyridoxal, or pyridoxamine) acts in the methylation cycle, and is essential for glycolysis and recharging the antioxidant glutathione. Low levels have been associated with migraine, chronic pain, and depression, especially in geriatric patients [31]. In an open trial in 52 hyperactive children, Vitamin B6 supplementation increased attention and reduced physical aggressivity [32]. Vitamin B12 (or cobalamin) is also part of the methylation cycle, and involved in the maintenance of myelin. Low levels have been associated with depression [31], irritability, fatigue, and diverse neuro-psychiatric abnormalities [33 34]. Low vitamin B9 (or folate) levels have been associated with weakness, depression, and behavioral disorders [34]. Dosages of our supplement are relatively high in B vitamins compared to the previous studies, as supplements containing higher doses of B vitamins were more effective for improving hostility and other stress-related mood states in a recent meta-analysis [35]. Moreover, in a dose-finding RCT study by Eussen et al. [36], only the supplementation daily doses of 647 to 1032 µg of cyanocobalamin (vitamin B12) was associated with normalization of mild vitamin B12 deficiency in older people.

Antioxidants (e.g., vitamins A, C and E; polyphenols)

Oxygen is required for metabolic activities, but also results in the production of free radicals (similar to the effects of tobacco smoke). These free radicals may impair lipid membranes, DNA, RNA, and normal cell function. Vitamin C is the best-known antioxidant. There is indirect evidence that oxidative stress in the brain contributes to mood and behavioral problems. We found that the level of dispositional optimism was associated with higher intakes of fruit and vegetables in the Zutphen study [37]. The potential effects of antioxidants on brain neurogenesis and plasticity may also be of importance in the role to prevent violent and aggressive behavior, but more studies in humans are needed. Vitamin A (or the active form retinol) is well known for its importance for vision. Deficiencies are rare in Western countries; they result from excessive alcohol use and rigorous dietary restrictions, and may lead to blindness. However, vitamin A also plays a functional role in the (development of the) brain. Vitamin E (tocopherols and tocotrienols) is formed by fat-soluble antioxidants with neuroprotective properties. Deficiency was associated with neuropathy, weakness, and retinal damage [38].

Other Vitamins and Minerals

Endogenous vitamin D results from dietary consumption (especially fatty fish and fortified foods) and endogenous production from cholesterol under the influence of sunlight (ultraviolet B) exposure. The biologically active metabolite 1,25(OH)2D is best known for its effects on calcium homeostasis, but also has important effects on the brain through its brain metabolism and receptors that are expressed in neuronal and glial cells (hippocampus, prefrontal cortex, hypothalamus, thalamus, and substantia nigra) [39]. Without sunlight exposure and an adequate vitamin D intake (> 600 IU or 15 µg per day) there is a high risk of deficiency. We showed in a pilot study that in groups of younger (<60 year old; n=33) and older (>60 year old; n=32) chronic psychiatric inpatients, 83% and 91% had mild to severe vitamin D deficiency (<50 nmol/L). Rodent studies suggest that vitamin D is involved in apoptosis of brain cells [40], dopamine and norepinephrine activity [41] as well as in social behavior [42]. Mice lacking functioning vitamin D receptors showed abnormal mothering, cannibalism, depressive-like behavior, but less aggressiveness compared to wild-type mice [42]. Choline is the precursor of the neurotransmitter acetylcholine. Low iron levels were associated with aggression and juvenile delinquency [43]. Small children at the age of 3 with zinc, iron, and vitamin B deficiencies exhibited greater antisocial, aggressive, and hyperactive behaviors 5, 8, and 14 years later [23] [44].

N-3 fatty acids

About 50% of the dry weight of the brain is constituted of lipids, and a large portion of these lipids consists of polyunsaturated fatty acids (PUFA). DHA is a major structural component of brain cells (thus is not used for energy), which acts together with EPA as precursors of anti-inflammatory and vasodilatory eicosanoids. Moreover, DHA is an essential part of phospholipids present in myelin and cell membranes, where it affects serotonergic action, inflammation, membrane fluidity, and membrane–protein interaction. Genomic studies have revealed effects of n-3 FA on gene expressions in rodent brains that are involved in ion channels, neuronal cell formation and function, signal transduction, synaptic plasticity, cytoskeleton, and energy metabolism [45]. Several lines of evidence suggest a relationship between serum levels of n-3 fatty acids DHA and EPA and aggressive behavior [46]. A study in rats showed that an n-3 deficient diet for 15 weeks reduced the brain DHA level by 40% and increased the level of aggression [47]. Dietary fatty fish or DHA was associated with less hostility [48]. In human volunteers, a double-blind placebo controlled study demonstrated that those 22 students who took DHA supplementation for 3 months showed less aggression associated with stressful conditions than 19 students using placebo [49]. In an RCT, 49 patients with recurrent self-harm were randomized to high dose EPA + DHA or placebo for 12 weeks, resulting in improvements in depression, suicidality and daily stresses, although

impulsivity, aggression and hostility did not significantly differ [50]. In 22 substance abusers, a high dose of EPA + DHA for 3 months significantly decreased anger and anxiety scores compared to placebo [51].

6.3 Summary of known and potential risks and benefits

See Supplementary Material 2 for an overview of product characteristics and specifications of Orthica Fish EPA Mini and Orthica Multi Energie and see Chapter 11 (STRUCTURED RISK ANALYSIS).

6.4 Description and justification of route of administration and dosage

Three supplement or placebo capsules will be taken by patients on a daily basis during six months (see Supplementary Material 2).

Ongoing studies

A recently finished trial is the PINUP (PrIson NUtrition Project) testing the effects of vitamins, minerals and essential fatty acids to reduce anti-social behavior in young offenders (16 to 21 years) in prison (ISRCTN41104834). This trial started in March 2009 and was expected to run for 4 years, and was funded by the Welcome Trust. Dosage in this study can be found in table 1. The main outcome parameters are violence, drug-related offences and incidents of self-harm. Another recently finished trial (The Swansea Trail) completed its treatment phase in July 2011 but not yet published. It included adult (age 18-35) male healthy volunteers to study whether aggression and impulsivity respond to multi-vitamins/minerals or fatty acid supplementation (NCT01558193 by David Benton, Professor of Psychology, Swansea University). Supplementation continued for 3 months, dosage in this study can be found in table 1. The main outcome parameters are impulsivity, aggression and frustration tests.

6.5 Dosages, dosage modifications and method of administration

Both supplements and placebos will be distributed by nursing staff as part of daily medication distribution procedures or through patients' Baxters, which are filled by the local pharmacist. Dosages will not be modified throughout the trial.

6.6 Preparation and labelling of Investigational Medicinal Product

Supplements will be prepared and labelled by Atrium Innovations Inc., producer of Orthica products.

6.7 Drug accountability

Supplements and placebo capsules will be labelled and distributed by Atrium Innovations Inc. (producer of Orthica products) to the participating institutions. Local pharmacies or nursing staff will confirm receipt and distribute supplements and placebo capsules to patients. In the event of unused supplements or placebo capsules, these will be collected, documented and subsequently destroyed by the local pharmacies or returned to the coordinating investigator.

7. METHODS

7.1 Study parameters/endpoints

7.1.1 Main study parameter/endpoint

The main parameter in this study is the number of aggressive incidents in each arm, registered with the SOAS-R [3]. As incidents may differ in severity and consequences, we make a distinction between minor (verbal aggression, threats, non-compliance with hospital rules, aggression towards objects, disinhibited [sexual] behaviour) and major (severe threats, fighting, assault on patients or staff, self-harm, suicide attempt) incidents. We carried out a pilot study to determine the prevalence of aggressive incidents among long-term psychiatric inpatients. This study yielded an estimate of 90 incidents per patient per year: 63 verbal aggression incidents, 8 incidents in which aggression was aimed at objects, 7 self-harm incidents, and 12 incidents in which physical aggression was aimed at others. We also monitored the time spent by nursing staff on each of these four types of incidents; verbal aggression took 80 minutes, aggression towards objects cost 77 minutes, self-harm cost 223 minutes, and physical aggression towards others cost 336 minutes per incidents. Based on these results, major incidents will be weighted by a factor 3.8.

7.1.2 Secondary study parameters/endpoints (if applicable)

Secondary parameters are:

- patient barriers and facilitators in the acceptation of nutritional supplements
- costs of time spent by staff members on aggression incidents and additional costs of incidents
- patient self-report aggression levels as measured with the AVL-AV [1]
- patient observer rated aggression levels as measured with the SDAS [2]
- patient observer rated affective symptoms as measured with the vCPRS [4]
- patient quality of life as measured with the WHOQL-bref [5]

7.1.3 Other study parameters (if applicable)

The following information will be collected at baseline and during follow-up and taken into account during analyses:

- patient nutritional status as measured in blood serum levels (to assess compliance)
- gender
- age
- diagnoses
- ward (*open* versus *closed*)
- medication
- legal measures
- psychotherapy
- institution
- height and weight (to calculate the body mass index [BMI])
- blood pressure and pulse frequency
- drug use
- social demographic information

7.2 Randomisation, blinding and treatment allocation

Patients will be randomised in a 1:1 ratio to either supplements (n=100) or placebo (n=100), using blocks of 12 patients to guarantee balanced groups sizes. Randomisation will be stratified for gender and ward-type (open or closed). Patients, nursing staff, psychiatrists, and research personnel will be blinded to treatment allocation. Concealment of treatment allocation will be ensured by a central computerized randomisation procedure, carried out by research staff not involved in the study (M.A. van den Hoorn, G.A.M. Corton). Randomisation can only be de-blinded by the research staff originally responsible for the randomisation procedure. This will be done once recruitment, data collection, and data cleaning are complete. In addition, individual codes can be broken whenever a serious adverse event

occurs for any of the participants; every six months, a comparison of adverse events in the active condition and the control condition will be made by a member of research staff not involved in this study (M.A. van den Hoorn, G.A.M. Corton). Blinding will be evaluated at the end of the trial by asking participants whether they think they received supplements or placebo.

7.3 Study procedures

7.3.1 Enrolment and short qualitative study

The patients will be recruited during the study start-up and inclusion phase from the long-stay units of the institutions involved in this study. Patients who meet inclusion- and exclusion criteria will be identified and given basic information by their treating psychiatrist or the local researcher through the patient information folder and in verbal communication. If the patient is interested, they can let the local investigator or their treating psychiatrist know, and they will inform the research team. After at least one week but within two weeks after identification, a (local) researcher will contact the patient to make an appointment for informed consent. Prior to this appointment, the treating psychiatrist will assess the patient's capacity to decide about participation. If there are doubts about the patients capacity to decide, or if the patient is deemed incapacitated, a legal representative (if applicable), an authorised person (if applicable), or a close relative will be present for informed consent. During this appointment, the study will be discussed further, questions will be answered, and written informed consent will be obtained.

7.3.2 Randomisation procedure, baseline assessment and intervention

Patients start with a 2 week run-in phase during which they will take three placebo capsules daily. After two weeks, patients will be contacted and the use of supplements will be evaluated using the short questionnaire on barriers and facilitators towards supplement use. If this evaluation is positive (patients are willing and able to take supplements on a daily basis), baseline information consisting of aggression scores (AVL-AV [self-report] [1], SDAS [observer rated] [2]), affective symptoms (vCPRS [observer rated] [4]), quality of life scores (WHOQL-bref [self-report] [5]), nutritional status (blood samples), and patient background information (BMI, blood pressure, medication and social demographic information) will be collected by either a researcher or a research nurse (t0). Patients are then randomised in a 1:1 ratio to either supplements (n=100) or placebo (n=100) (see section 5 investigational product). Randomisation will be stratified for gender and ward-type (open or closed) and will be carried out in blocks of 12 patients to guarantee balanced group sizes. Randomisation will

be blind to patients, nurses, psychiatrists, outcome assessors and data-analysts. Concealment of treatment allocation will be ensured by a central computerized randomisation procedure, carried out by research staff not involved in the study (M.A. van den Hoorn, G.A.M. Corton). After randomisation, daily administration of three capsules (either supplements or placebo) will start. Capsules will be administered daily by nursing staff as part of regular medication distribution, or in Baxters prepared by the pharmacist. The intervention will continue for six months after randomisation.

7.3.3 Follow-up assessments

Throughout the intervention period, aggressive incidents will be registered using the SOAS-R [3]. At two weeks after baseline (t1), patient aggression scores will be assessed with the SDAS [2] (observer rated). At two months post-baseline (t2), follow-up data consisting of aggression scores (AVL-AV [1] [self-report], SDAS [2] [observer rated]), affective symptoms (vCPRS [4] [observer rated]), and quality of life scores (WHOQL-bref [5] [self-report]), will be collected by either a researcher or a research nurse. During the last week of the intervention period (t3), final data collection will take place, consisting of blood samples, aggression scores (AVL-AV [1] [self-report], SDAS [2] [observer rated]), affective symptoms (vCPRS [4] [observer rated]), and quality of life scores (WHOQL-bref [5] [self-report]).

7.3.4 Data collection

7.3.4.1 Aggressive incidents

For the whole duration of the intervention period (six months), aggressive incidents involving participating patients will be registered by nursing staff using the SOAS-R [3].

7.3.4.2 Blood samples

At baseline and end of intervention (after 6 months) blood samples will be collected to determine nutritional status to monitor compliance. Two tubes will be collected (1 serum, and 1 EDTA), and samples will be stored at –80ºC and analysed for Vitamin A, E, B1, B6, B12, and D, folate and iron, as well as a fatty acid spectrum to yield n-3 FA levels (ALA, EPA, and DHA). As other study data, blood samples will be stored over a period of 15 years after finalisation of the research report. The main purpose of the collection of blood samples within this study is to monitor compliance. However, future analyses in line with the original research question may be necessary. As specified in the patient information letter and informed consent, patients can give permission for these additional analyses, providing they are in line with the original research question, and do not concern outcomes that could hold clinical relevance for the patient. Should any additional, new, research questions emerge

within the 15-year storage period, patients will be approached to provide additional informed consent for these analyses. This procedure is explicated in the patient information folder and has been included in the informed consent form.

7.3.4.2 Aggression scores self-report

Patient feelings of aggression and agitation will be assessed at baseline, at two month follow-up and at end of intervention using the AVL-AV [1], a 12-item questionnaire measuring aggression.

7.3.4.3 Aggression scores observer rated

Patient observed aggression and agitation levels will be assessed at baseline, at two month follow-up and at end of intervention using the SDAS [2], an 11-item observer rated questionnaire measuring aggression and hostility.

7.3.4.4 Affective symptoms observer rated

Presence and severity of a wide range of psychiatric symptoms will be assessed at baseline, two-month follow-up and at the end of intervention using the vCPRS [4], a 25-item observer rated questionnaire measuring common affective symptoms.

7.3.4.5 Quality of life self-report

Patient quality of life will be assessed at baseline, at two month follow-up and at end of intervention using the WHOQL-bref [5], a 26 item self-report questionnaire measuring quality of life.

7.4 Withdrawal of individual subjects

Participants can leave the study at any time for any reason if they wish to do so without any consequences. They do not have to state their reason for leaving the study. The investigator or treating psychiatrists can decide to withdraw any participant from the study for urgent medical reasons.

7.5 Replacement of individual subjects after withdrawal

As this is a pragmatic trial, primary analysis will be intention to treat (ITT) and participants will not be replaced after withdrawal.

7.6 Follow-up of subjects withdrawn from treatment

As we expect the only reason for withdrawal from the study will be the refusal of patients to continue participation in the study, data collection for these patients will cease.

7.7 Premature termination of the study

In case of significantly increased incidence of serious side-effects in patients treated with the active supplements, the study will be ended. Adverse Events (AE's) and Serious Adverse Events (SAE's) will be monitored (see 8.2). As these are widely used dietary supplements for which safety has been established, such an occurrence is highly unlikely.

8. SAFETY REPORTING

8.1 Section 10 WMO event

In accordance to section 10, subsection 1, of the WMO, the investigator will inform the subjects and the reviewing accredited METC if anything occurs, on the basis of which it appears that the disadvantages of participation may be significantly greater than was foreseen in the research proposal. The study will be suspended pending further review by the accredited METC, except insofar as suspension would jeopardise the subjects' health. The investigator will take care that all subjects are kept informed.

8.2 AEs, SAEs and SUSARs

8.2.1 Adverse events (AEs)

Adverse events are defined as any undesirable experience occurring to a subject during the study, whether or not considered related to Orthica Multi Energie and Fish EPA Mini. All adverse events reported spontaneously by the subject or observed by the investigator or his staff will be recorded and reported through the webportal ToetsingOnline.

8.2.2 Serious adverse events (SAEs)

A serious adverse event is any untoward medical occurrence or effect that at any dose:

- results in death;
- is life threatening (at the time of the event);
- requires hospitalisation or prolongation of existing inpatients' hospitalisation;
- results in persistent or significant disability or incapacity;
- is a congenital anomaly or birth defect;

- Any other important medical event that may not result in death, be life threatening, or require hospitalization, may be considered a serious adverse experience when, based upon appropriate medical judgement, the event may jeopardize the subject or may require an intervention to prevent one of the outcomes listed above.

The local investigator will monitor and register the event and will inform the coordinating investigator who will inform the sponsor and principal investigator, as well as M.A. van den Hoorn and G.A.M. Corton.

The sponsor will assign the coordinating investigator to report the SAEs through the web portal *ToetsingOnline* to the accredited METC that approved the protocol, within 15 days after the sponsor has first knowledge of the serious adverse reactions.

SAEs that result in death or are life threatening should be reported expedited. The expedited reporting will occur not later than 7 days after the responsible investigator has first knowledge of the adverse reaction. This is for a preliminary report with another 8 days for completion of the report.

8.3 Follow-up of adverse events

All AEs will be followed until they have abated, or until a stable situation has been reached. Depending on the event, follow up may require additional tests or medical procedures as indicated, and/or referral to the general physician or a medical specialist. SAEs will be reported till the end of study within the Netherlands, as defined in the protocol.

9. STATISTICAL ANALYSIS

9.1 Primary study parameter

The primary parameter will be the number of aggressive incidents in each arm. Incidents will be registered by nurses using the SOAS-R. As incidents may differ in severity and consequences, we make a distinction between minor (verbal aggression, threats, non-compliance with hospital rules, aggression towards objects, disinhibited [sexual] behaviour) and major (severe threats, fighting, assault on patients or staff, self-harm, suicide attempt) incidents. As our pilot study demonstrated that on average, nursing staff spent 80 minutes on verbal aggression, 77 minutes on aggression towards objects, 223 minutes on self-harm incidents, and 336 minutes on physical aggression towards others; major incidents will be

weighted by a factor 3.8. The number of incidents in each arm will be compared following the intention to treat approach. Univariable analysis will be performed using t-test (primary analysis). Multivariable analyses adjusting for age, diagnoses, medication, and institution will be performed using multiple regression. Analyses will first be carried out using blinded treatment codes A and B (without knowledge of active treatment content; triple blind analysis). No interim analyses are planned.

9.2 Secondary study parameters

- *Costs of* time *spent by staff members on aggression incidents and additional costs of incidents*. Incidents as well as net costs are expected to decrease. In a cost-effectiveness analysis the difference in intervention costs will be compared to the difference in the primary outcome measure of the study (intervention costs per prevented incident, using net benefit analysis). In addition, net societal costs will be compared in a cost minimization analysis. Analyses will be performed in accordance with the pharmacoeconomic guidelines, using a one-year time horizon without discounting. Estimated costs will include the intervention itself (market price of supplements, with administration time) and costs associated with minor and major incidents. A separate cost price analysis will be performed to trace the average consequences and costs per minor and major incident (including direct staff time, coerced medication, emergency department visits, involvement of police, physicians and guards). Budget impact will be evaluated from the perspective of society, BKZ/Justice, health insurer and the institutions for long-term psychiatric care. Currently there is no budgetary compensation for supplementation of vitamins, minerals, and n-3FA. The costs of supplementation will burden the institutes, who will be compensated by savings from prevented incidents.
- *Patient self-report aggression levels as measured with the AVL-AV* [1]*, Patient observer rated aggression levels as measured with the SDAS* [2]*, Patient observer rated psychiatric symptoms as measured with the vCPRS* [4]*, Patient quality of life as measured with the WHOQL-bref* [5]*.* Changes from pre- to post intervention in each arm will be analysed for patient nutritional status, subjective aggression levels, observed aggression levels, observed affective symptoms and quality of life, using mixed model/ multiple regression analysis. All analyses will be both ITT (primary analysis) and per protocol (PP; secondary analysis). In case of missing data we will use last observation carried forward for the ITT analyses.

10. ETHICAL CONSIDERATIONS

10.1 Regulation statement

The study will be conducted according to the principles of the Declaration of Helsinki (version October 2013) and in accordance with the Medical Research Involving Human Subjects Act (WMO).

10.2 Recruitment and consent

The 200 patients will be recruited from the long-stay units of the institutions involved in this study. Patients who meet inclusion- and exclusion criteria will be identified and given basic information by their treating psychiatrist or the local researcher through the patient letter, patient information folder, accompanying flyer, and in verbal communication. If the patient is interested, they can let the local investigator or their treating psychiatrist know, who will inform the research team. After at least one week and within two weeks after identification, a researcher will contact the patient to make an appointment for informed consent. Prior to this appointment, the treating psychiatrist will assess the patient’s ability to decide about participation. If there are doubts about the patients capacity to decide, or if the patient is deemed incapacitated, a close relative or, if applicable, legal representative will be present. During this appointment, the study will be discussed further, questions will be answered, and written informed consent will be obtained. The informed consent form will be signed in duplicate, one copy will be reported in the Case Report Form (CRF), the second copy will be given to the participant.

10.3 Objection by minors or incapacitated subjects (if applicable)

Although the majority of patients residing at institutions for long-term psychiatric care is expected to be able to decide about participating in the study, some may be (partially) incompetent to decide whether or not to participate in the study, in addition, some may be legally incompetent. We firmly believe however, that the research question cannot be answered in any other patient group and that there is a chance that participation in the study may benefit the research patient (art. 4 lid 1 WMO). Of course every care will be taken in the informed consent procedure to explain the study in a way that is understandable for the long-term psychiatric inpatient. Also, the treating psychiatrist will assess the patient’s ability to decide about participation prior to taking informed consent. If there are doubts about the patients capacity to decide, or if the patient is deemed incapacitated, we will involve the legal representative (if applicable), the authorised person (if applicable), or a close relative who

might act as a proxy (surrogate) decision maker. Close relatives will be considered in the order specified in the 'model informatiebrief' provided by ccmo: husband/wife; partner; life companion; parents; available adult children or available adult siblings. Individual considerations like expected willingness to undergo study procedures (e.g. questionnaires and collecting of blood samples) will be taken into account when the patient is less able to make the decision to participate. During the study, well-being and willingness of (legally) incompetent patients, as shown in reactions of patients, will be taken into account as important criteria for deciding whether or not to continue.

10.4 Benefits and risks assessment, group relatedness

Benefits

This study will provide information about effects of vitamin-, mineral-, and n-3FA supplementation on aggressive behaviour and well-being in long-term psychiatric inpatients that is not available at present. Potential benefits of participating in this study are a direct and indirect increase in quality of life through improved nutrition as well as reduction of aggressive incidents. Participants will receive compensation for participating in the study (see 11.6 incentives).

Burden

The burden associated with participation is minimal. Aggressive incidents are often already monitored and registered by psychiatric nurses as part of usual care. This registration procedure will be standardized across institutions by using the SOAS-R [3]. As the SOAS-R is a registration tool filled in by nursing staff, it does not pose a burden for patients. Patient nutritional status will be determined through blood samples collected pre- and post-intervention. Patients' aggressive feelings and attitudes; general affective symptoms, and quality of life will be measured using the AVL-AV [1], the SDAS [2] (observer rated, filled in by nurses), the vCPRS [4] (observer rated), and the WHOQL-bref [5] pre-intervention, two months after start of intervention and post-intervention. The SDAS will also be administered 2 weeks after the start of the intervention. In order to further increase study feasibility, a short survey concerning patients' attitudes towards nutritional supplements will be developed from a series of 5 to 10 semi-structured interviews, and administered to all participants after the run in phase.

Risk assessment

The use of the supplements Orthica Multi Energie and Orthica Fish EPA Mini has not been associated with any risks (see SPC). The risks are therefore negligible. The risk of blood collection is that of a small local hematoma, feeling light headed, fainting or local infection.

Group relatedness

As this is a pragmatic trial in which the main objective is to determine whether the use of supplements has the potential to reduce the number of aggressive incidents in chronic psychiatric inpatients, it is imperative to involve these patients. We firmly believe that the research question cannot be answered in any other patient group or healthy population and that there is a chance that participation in the study may benefit the research patient.

10.5 Compensation for injury

The sponsor wishes to obtain dispensation from the statutory obligation to provide insurance, because participating in the study is associated with negligible risks. The use of the supplements Orthica Multi Energie and Orthica Fish EPA Mini has not been associated with important risks.

The LUMC has a liability insurance which is in accordance with article 7, subsection 6 of the WMO.

10.6 Incentives

Subjects will receive compensation for participating in the three points of data collection that require active participation by filling in questionnaires (t0, t2 and t3). In institutions for long-term psychiatric care, daily activity programmes exist in which patients are rewarded. In close consultation with the institutions involved in this study, we will devise a compensation scheme that closely follows existing reward systems and existing reward rates, which are around €2,50 per hour, or propose a small gift of equal value. As data collection at t0, t2 and t3 is expected to take approximately one hour per session, compensation will amount to €7,50 for the entire study per participant. Participants will be rewarded for each questionnaire session that has been started, completion of questionnaires is not required for compensation.

11. ADMINISTRATIVE ASPECTS, MONITORING AND PUBLICATION

11.1 Handling and storage of data and documents

All data collected in this study will be stored anonymously and handled confidentially. In order to ensure anonymity, all participants will receive a random participant number, all data will be stored under this participant number, and only the number will be used in further

analysis. Only the principal investigator (E.J. Giltay) and the coordinating investigator (N. Rius Ottenheim) will have access to the key for these codes and the original documents. A member of research-staff not involved in this study (M.A. van den Hoorn, G.A.M. Corton) will have access to the key for the codes in order to identify participants in case of adverse events that may be related to supplement use. Data will either be entered directly in the CRF of each participant or filled in on specific forms. Data from forms will be collected by the coordinating researcher and entered into the database. The database and data managing unit will be based at the department of psychiatry of the LUMC. The originals of all source documents as well as collected blood samples will be stored for a period of 15 years after finalization of the research report. Handling of data will follow Dutch legal requirements according to CCMO, WGBO and WBP. Participants have voluntarily approved of this procedure by signing the informed consent.

11.2 Amendments and add-on studies

Amendments are changes made to the research after a favourable opinion by the accredited METC has been given. All amendments and add-on studies will be notified to the METC that gave a favourable opinion. Pilot studies investigating the prevalence of aggression in institutions for long-term psychiatric care and the feasibility of administration of a food frequency questionnaire (FFQ) have already received a declaration of “no objection” of the LUMC METC and have been planned and carried out.

11.3 Annual progress report

The sponsor/investigator will submit a summary of the progress of the trial to the accredited METC once a year. Information will be provided on the date of inclusion of the first subject, numbers of subjects included and numbers of subjects that have completed the trial, serious adverse events/ serious adverse reactions, other problems, and amendments.

11.4 End of study report

The investigator will notify the accredited METC of the end of the study within a period of 8 weeks. The end of the study is defined as the last patient’s last measurement session.
In case the study is ended prematurely, the investigator will notify the accredited METC, including the reasons for the premature termination.

Within one year after the end of the study, the investigator/sponsor will submit a final study

report with the results of the study, including any publications/abstracts of the study, to the accredited METC.

11.5 Public disclosure and publication policy

The identity of the participants will not be disclosed in any way in publications. All results will be published in peer-reviewed national and international medical journals and presented at national and international conferences. The study will be registered in a public trial register before inclusion of the first patient. The names of the LUMC main investigators will be listed as authors on the main publication. These will be followed by the text ‘for the Diet and Aggression Trial.’ This is a so-called custom group authorship that lists individual names, followed by the group's name. Other names will be listed under "collaborators” in the following order:

• Steering Committee, naming all participants
• Scientific and Organizing Secretariat, naming all participants
• Diet and Aggression Trial Centers: Naming all participating centers and their participating main psychiatrist after the statement: "We thank the participating psychiatrists and their staff for their excellent cooperation."
• Funding / Support: 'The Diet and Aggression Trial is financially supported by a grant from ZonMw, the Dutch Organization for Health Research and Development (836031016).'
• 'Trial supplements were provided by Orthica, MCO Health, Almere, The Netherlands.'

For participating institutions, this means that, in principle, one institution is called by one collaborator. An exception is made when a participating institution provides 10 participants or more for the study, in which case one researcher will be eligible for co-authorship from that institution. The institution can decide who this person will be. This researcher will be invited to work as an author on the paper. To qualify for authorship, an individual must have participated sufficiently in the work to take public responsibility for all or part of the content, given final approval of the submitted version, and made substantive intellectual contributions to the submitted work in the form of: 1) conception and design, and/or acquisition of data, and/or analysis of data; and 2) drafting the article, and/or revising it critically for important intellectual content.

12. STRUCTURED RISK ANALYSIS

12.1 Potential issues of concern

In the RCTs among 71 incarcerated juvenile (13-17 year old) delinquents and 468 disciplined schoolchildren (6-12 year old) [11 12], vitamins and minerals were supplemented (but no n-3 FA). There is no mention of any side effects in these two studies. In the RCT by Gesch *et al.* [13] 231 adult (>18 year old) participants were recruited from prisons. The average time on supplementation was 143 days for the active compound. None of the subjects were withdrawn from the study as a result of side effects. Moreover, the institution's senior medical officer reported no adverse reactions to supplementation. In the RCT by Zaalberg *et al.* [14] among 326 participants from prisons, lunchtime was chosen as the moment of administration to minimize possible side effects such as nausea and belching. Participants received the supplements for at least one month and a maximum of 3 months. There was no mentioning of nausea, belching or other side effects in the study.

In a safety study using over 12 publications from 6 datasets including psychiatric patients [52], no clinically meaningful abnormal laboratory values were found upon supplementation with a mix of 36 different vitamins and minerals. The complex micronutrient formula used is called EMPowerplus (EMP+). In the reports, there was not a single reported occurrence of a clinically meaningful negative outcome/effect or an abnormal blood test that could be attributed to toxicity. There were only minor and transitory adverse events, such as headache and gastrointestinal problems (i.e., nausea, vomiting, and diarrhoea). The study populations included adults with bipolar disorder and obsessive-compulsive disorder, no adverse events occurred in these patient groups. Nevertheless, it was recommended that side effects should be monitored closely, as complex interactions with medication may require adjustments in doses in' non-healthy populations'. Moreover, a dose that is safe for one group may be harmful to others. It is also recommended not to take the formula on an empty stomach.

Based on reports of the Food and Agriculture Organization (FAO), the WHO, and authoritative bodies in the European Union, United Kingdom, and United States, there is a normal homeostatic range for vitamins and minerals (U shaped relationship), and the width of the range dependents on the nutrient [53]. The oxidized metabolites of retinol and beta-carotene (vitamin A) are known to be teratogenic and associated with congenital and bone abnormalities in humans, but our dose is substantially lower than the maximum safe dose and more in the range of the Recommended Daily Amount (RDA). As concomitant use of several different supplement products, individually containing RDA levels of nutrients, could lead to a total intake above upper levels, we are reluctant to include patients who already use supplements. However, psychiatric patients often already use vitamin B1 (thiamine) and/or vitamin D as part of their regular treatment. Therefore, we will not include patients who already use supplements, except for patients who use thiamine or vitamin D. The additional dose for the intervention group in combination with their regular dose will not involve any

health risks[54]. All in all, side effects are expected to be mild, but will be closely monitored, as is advised.

13. Reference List

(1) Hornsveld RHJ, Muris P, Kraaimaat FW, Meesters C. Psychometric Properties of the Aggression Questionnaire in Dutch Violent Forensic Psychiatric Patients and Secondary Vocational Students. *Assessment* 2009 June;16(2):181-92.

(2) Nijman HLI, Muris P, Merckelbach HLGJ, Palmstierna T, Wistedt B, Vos AM, van Rixtel A, Allertz W. The Staff Observation Aggression Scale-revised (SOAS-R). *Aggressive Behavior* 1999;25(3):197-209.

(3) Nijman HLI, Muris P, Merckelbach HLGJ, Palmstierna T, Wistedt B, Vos AM, van Rixtel A, Allertz W. The Staff Observation Aggression Scale-revised (SOAS-R). *Aggressive Behavior* 1999;25(3):197-209.

(4) Asberg M, Schalling D. Construction of A New Psychiatric Rating Instrument, the Comprehensive Psychopathological Rating-Scale (Cprs). *Progress in Neuro-Psychopharmacology* 1979;3(4):405-12.

(5) De Vries J, Van Heck GL. De Nederlandse versie van de WHOQOL-100 [The Dutch version of the WHOQOL-100]. *Tilburg University, Tilburg* 1995.

(6) Knispel A, Haggenburg ME, van Hoof F. Landelijke Monitor Intramurale GGZ: Eerste meting - 2012. Utrecht, The Netherlands: Trimbos Instituut; Netherlands Institute of Mental health and Addiction; 2013.

(7) Bowers L, Stewart D, Papadopoulos C, Dack C, Ross J, Khanom H, Jeffery D. Inpatient violence and aggression: a literature review. Kings College London: Section of Mental Health Nursing; Health Service and Population Research; Institute of Psychiatry; 2011.

(8) Nijman H, Bowers L, Oud N, Jansen G. Psychiatric nurses' experiences with inpatient aggression. *Aggressive Behavior* 2005;31(3):217-27.

(9) Arnetz JE. The Violent Incident Form (VIF): a practical instrument for the registration of violent incidents in the health care workplace. *Work and Stress* 1998 January;12(1):17-28.

(10) Daffern M, Howells K. Psychiatric inpatient aggression - A review of structural and functional assessment approaches. *Aggression and Violent Behavior* 2002 September;7(5):477-97.

(11) Schoenthaler S, Amos S, Doraz W, Kelly M-A, Muedeking G, Wakefield J. The effect of randomized vitamin-mineral supplementation on violent and non-violent antisocial behavior among incarcerated juveniles. *J Nutr Environ Med* 1997;9:343-52.

(12) Schoenthaler SJ, Bier ID. The effect of vitamin-mineral supplementation on juvenile delinquency among American schoolchildren: a randomized, double-blind placebo-controlled trial. *J Altern Complement Med* 2000 February;6(1):7-17.

(13) Gesch CB, Hammond SM, Hampson SE, Eves A, Crowder MJ. Influence of supplementary vitamins, minerals and essential fatty acids on the antisocial behaviour of young adult prisoners. Randomised, placebo-controlled trial. *Br J Psychiatry* 2002 July;181:22-8.

(14) Zaalberg A, Nijman H, Bulten E, Stroosma L, van der Staak C. Effects of Nutritional Supplements on Aggression, Rule-Breaking, and Psychopathology Among Young Adult Prisoners. *Aggressive Behavior* 2010 March;36(2):117-26.

(15) Legare N, Brosseau E, Joyal CC. Omega-3 and violence in schizophrenia. *Schizophrenia Research* 2007 November;96(1-3):269.

(16) Francis JJ, Johnston M, Robertson C, Glidewell L, Entwistle V, Eccles MP, Grimshaw JM. What is an adequate sample size? Operationalising data saturation for theory-based interview studies. *Psychology & Health* 2010;25(10):1229-45.

(17) Grol R, Wensing M, Eccles MP. *Improving patient care*. Edinburgh: Elsevier; 2005.

(18) Gray GE, Gray LK. Nutritional Aspects of Psychiatric-Disorders. *Journal of the American Dietetic Association* 1989 October;89(10):1492-8.

(19) Higashi K, Medic G, Littlewood KJ, Diez T, Granstrom O, De HM. Medication adherence in schizophrenia: factors influencing adherence and consequences of nonadherence, a systematic literature review. *Ther Adv Psychopharmacol* 2013 August;3(4):200-18.

(20) Benton D. The impact of diet on anti-social, violent and criminal behaviour. *Neuroscience and Biobehavioral Reviews* 2007;31(5):752-74.

(21) Parletta N, Milte CM, Meyer BJ. Nutritional modulation of cognitive function and mental health. *Journal of Nutritional Biochemistry* 2013 May;24(5):725-43.

(22) Neugebauer R, Hoek HW, Susser E. Prenatal exposure to wartime famine and development of antisocial personality disorder in early adulthood. *Jama-Journal of the American Medical Association* 1999 August 4;282(5):455-62.

(23) Liu JH, Raine A, Venables PH, Dalais C, Mednick SA. Malnutrition at age 3 years and lower cognitive ability at age 11 years - Independence from psychosocial adversity. *Archives of Pediatrics & Adolescent Medicine* 2003 June;157(6):593-600.

(24) Mookhoek EJ, Colon EJ. [Nutritional status of elderly patients at admission to a general psychiatric hospital. An inventory]. *Tijdschr Gerontol Geriatr* 1992 August;23(4):127-31.

(25) Sloane PD, Ivey J, Helton M, Barrick AL, Cerna A. Nutritional issues in long-term care. *Journal of the American Medical Directors Association* 2008 September;9(7):476-85.

(26) Popova NK. From gene to aggressive behavior: the role of brain serotonin. *Neurosci Behav Physiol* 2008 June;38(5):471-5.

(27) Siever LJ. Neurobiology of aggression and violence. *American Journal of Psychiatry* 2008 April;165(4):429-42.

(28) Bjork JM, Dougherty DM, Moeller FG, Cherek DR, Swann AC. The effects of tryptophan depletion and loading on laboratory aggression in men: time course and a food-restricted control. *Psychopharmacology* 1999 February;142(1):24-30.

(29) Coccaro EF, Kavoussi RJ. Fluoxetine and impulsive aggressive behavior in personality-disordered subjects. *Archives of General Psychiatry* 1997 December;54(12):1081-8.

(30) Coccaro EF, Lee RJ, Kavoussi RJ. A Double-Blind, Randomized, Placebo-Controlled Trial of Fluoxetine in Patients With Intermittent Explosive Disorder. *Journal of Clinical Psychiatry* 2009 May;70(5):653-62.

(31) Skarupski KA, Tangney C, Li H, Ouyang BC, Evans DA, Morris MC. Longitudinal association of vitamin B-6, folate, and vitamin B-12 with depressive symptoms among older adults over time. *American Journal of Clinical Nutrition* 2010 August;92(2):330-5.

(32) Mousain-Bosc M, Roche M, Rapin J, Bali JP. Magnesium VitB6 intake reduces central nervous system hyperexcitability in children. *Journal of the American College of Nutrition* 2004 October;23(5):545S-8S.

(33) Lindenbaum J, Healton EB, Savage DG, Brust JCM, Garrett TJ, Podell ER, Marcell PD, Stabler SP, Allen RH. Neuropsychiatric Disorders Caused by Cobalamin Deficiency in the Absence of Anemia Or Macrocytosis. *New England Journal of Medicine* 1988 June 30;318(26):1720-8.

(34) Coppen A, Bolander-Gouaille C. Treatment of depression: time to consider folic acid and vitamin B-12. *Journal of Psychopharmacology* 2005 January;19(1):59-65.

(35) Long SJ, Benton D. Effects of Vitamin and Mineral Supplementation on Stress, Mild Psychiatric Symptoms, and Mood in Nonclinical Samples: A Meta-Analysis. *Psychosomatic Medicine* 2013 February;75(2):144-53.

(36) Eussen SJ, de Groot LC, Joosten LW, Bloo RJ, Clarke R, Ueland PM, Schneede J, Blom HJ, Hoefnagels WH, van Staveren WA. Effect of oral vitamin B-12 with or without folic acid on cognitive function in older people with mild vitamin B-12 deficiency: a randomized, placebo-controlled trial. *American Journal of Clinical Nutrition* 2006 August;84(2):361-70.

(37) Giltay EJ, Geleijnse JM, Zitman FG, Buijsse B, Kromhout D. Lifestyle and dietary correlates of dispositional optimism in men: The Zutphen Elderly Study. *Journal of Psychosomatic Research* 2007 November;63(5):483-90.

(38) Khanna S, Roy S, Parinandi NL, Maurer M, Sen CK. Characterization of the potent neuroprotective properties of the natural vitamin E alpha-tocotrienol. *Journal of Neurochemistry* 2006 September;98(5):1474-86.

(39) Garcion E, Wion-Barbot N, Montero-Menei CN, Berger F, Wion D. New clues about vitamin D functions in the nervous system. *Trends in Endocrinology and Metabolism* 2002 April;13(3):100-5.

(40) Feron F, Burne THJ, Brown J, Smith E, McGrath JJ, Mackay-Sim A, Eyles DW. Developmental Vitamin D-3 deficiency alters the adult rat brain. *Brain Research Bulletin* 2005 March 15;65(2):141-8.

(41) Baksi SN, Hughes MJ. Anti-Dopaminergic Effects of Estradiol and Tamoxifen in the Brain of Immature Rabbits. *Federation Proceedings* 1982;41(4):1064.

(42) Kalueff AV, Keisala T, Minasyan A, Kuuslahti M, Miettinen S, Tuohimaa P. Behavioural anomalies in mice evoked by "Tokyo" disruption of the Vitamin D receptor gene. *Neuroscience Research* 2006 April;54(4):254-60.

(43) Rosen GM, Deinard AS, Schwartz S, Smith C, Stephenson B, Grabenstein B. Iron deficiency among incarcerated juvenile delinquents. *Journal of Adolescent Health Care* 1985;6(6):419-23.

(44) Raine A, Mellingen K, Liu JH, Venables P, Mednick SA. Effects of environmental enrichment at ages 3-5 years on schizotypal personality and antisocial

behavior at ages 17 and 23 years. *American Journal of Psychiatry* 2003 September;160(9):1627-35.

(45) Hammamieh R, Chakraborty N, Gautam A, Miller SA, Muhie S, Meyerhoff J, Jett M. Transcriptomic Analysis of the Effects of a Fish Oil Enriched Diet on Murine Brains. *Plos One* 2014 March 14;9(3).

(46) Hibbeln JR, Ferguson TA, Blasbalg TL. Omega-3 fatty acid deficiencies in neurodevelopment, aggression and autonomic dysregulation: Opportunities for intervention. *International Review of Psychiatry* 2006 April;18(2):107-18.

(47) Demar JC, Ma KZ, Bell JM, Igarashi M, Greenstein D, Rapoport SI. One generation of n-3 polyunsaturated fatty acid deprivation increases depression and aggression test scores in rats. *Journal of Lipid Research* 2006 January;47(1):172-80.

(48) Iribarren C, Reed DM, Wergowske G, Burchfiel CM, Dwyer JH. Serum cholesterol level and mortality due to suicide and trauma in the Honolulu Heart Program. *Arch Intern Med* 1995 April 10;155(7):695-700.

(49) Hamazaki T, Sawazaki S, Itomura M, Asaoka E, Nagao Y, Nishimura N, Yazawa K, Kuwamori T, Kobayashi M. The effect of docosahexaenoic acid on aggression in young adults - A placebo-controlled double-blind study. *Journal of Clinical Investigation* 1996 February 15;97(4):1129-33.

(50) Hallahan B, Hibbeln JR, Davis JM, Garland MR. Omega-3 fatty acid supplementation in patients with recurrent self-harm - Single-centre double-blind randomised controlled trial. *British Journal of Psychiatry* 2007 February;190:118-22.

(51) Buydens-Branch, Branchey M, Hibbeln JR. Associations between increases in plasma n-3 polyunsaturated fatty acids following supplementation and decreases in anger and anxiety in substance abusers. *Progress in Neuro-Psychopharmacology & Biological Psychiatry* 2008 February 15;32(2):568-75.

(52) Simpson JS, Crawford SG, Goldstein ET, Field C, Burgess E, Kaplan BJ. Systematic review of safety and tolerability of a complex micronutrient formula used in mental health. *BMC Psychiatry* 2011;11:62.

(53) Mulholland CA, Benford DJ. What is known about the safety of multivitamin-multimineral supplements for the generally healthy population? Theoretical basis for harm. *American Journal of Clinical Nutrition* 2007 January;85(1):318S-22S.

(54) European Food Safety Authority: Scientific Committee on Food. (2006). *Tolerable upper intake levels for vitamins and minerals.* Parma, Italy.
